# Medicinal Plant Extracts against Cardiometabolic Risk Factors Associated with Obesity: Molecular Mechanisms and Therapeutic Targets

**DOI:** 10.3390/ph17070967

**Published:** 2024-07-21

**Authors:** Jorge Gutiérrez-Cuevas, Daniel López-Cifuentes, Ana Sandoval-Rodriguez, Jesús García-Bañuelos, Juan Armendariz-Borunda

**Affiliations:** 1 Department of Molecular Biology and Genomics, Institute for Molecular Biology in Medicine and Gene Therapy, University Center of Health Sciences, University of Guadalajara, Guadalajara 44340, Jalisco, Mexico; daniel.2cifuentes@gmail.com (D.L.-C.); anasol44@hotmail.com (A.S.-R.); armdbo@gmail.com (J.A.-B.); 2 Doctorate in Sciences in Molecular Biology in Medicine, University Center of Health Sciences, University of Guadalajara, Guadalajara 44340, Jalisco, Mexico; 3 Escuela de Medicina y Ciencias de la Salud (EMCS), Tecnologico de Monterrey, Campus Guadalajara, Zapopan 45201, Jalisco, Mexico

**Keywords:** obesity, cardiometabolic risk factors, herbs, plants, extracts, molecular mechanisms, therapeutic targets

## Abstract

Obesity has increasingly become a worldwide epidemic, as demonstrated by epidemiological and clinical studies. Obesity may lead to the development of a broad spectrum of cardiovascular diseases (CVDs), such as coronary heart disease, hypertension, heart failure, cerebrovascular disease, atrial fibrillation, ventricular arrhythmias, and sudden cardiac death. In addition to hypertension, there are other cardiometabolic risk factors (CRFs) such as visceral adiposity, dyslipidemia, insulin resistance, diabetes, elevated levels of fibrinogen and C-reactive protein, and others, all of which increase the risk of CVD events. The mechanisms involved between obesity and CVD mainly include insulin resistance, oxidative stress, inflammation, and adipokine dysregulation, which cause maladaptive structural and functional alterations of the heart, particularly left-ventricular remodeling and diastolic dysfunction. Natural products of plants provide a diversity of nutrients and different bioactive compounds, including phenolics, flavonoids, terpenoids, carotenoids, anthocyanins, vitamins, minerals, fibers, and others, which possess a wide range of biological activities including antihypertensive, antilipidemic, antidiabetic, and other activities, thus conferring cardiometabolic benefits. In this review, we discuss the main therapeutic interventions using extracts from herbs and plants in preclinical and clinical trials with protective properties targeting CRFs. Molecular mechanisms and therapeutic targets of herb and plant extracts for the prevention and treatment of CRFs are also reviewed.

## 1. Introduction

Obesity is a worldwide public health problem and is a chronic non-transmissible disease whose incidence has been dramatically increasing around the world. This chronic metabolic disease affects distinct age, ethnic and social classes, and has a tremendous impact on the economy and quality of life. Obesity and being overweight are the fifth main causes of deaths globally [1,2]. According to the World Health Organization (WHO), overweight people are defined as having a body mass index (BMI) > 25, while people with a BMI > 30 are considered obese [1]. The pathophysiology of obesity implicates a combination of genetic/epigenetic, nutritional, and environmental factors that promote a chronic positive energy balance and expansion of body fat mass, specially of white adipose tissue (WAT) in visceral fat depots [1,2]. In obese people, WAT plays a key role in secreting lipolysis or lipid synthesis hormones, including inflammatory cytokines that regulate lipid metabolism, and promotes a low-grade chronic inflammation that has the potential to activate insulin resistance and endothelial dysfunction [1,3,4,5]. An excessive accumulation of fat in WAT, in addition to obesity, can also cause metabolic disorders such as dyslipidemia, adipocyte dysfunction, metabolic syndrome, hypertension, type 2 diabetes (T2D), metabolic dysfunction-associated steatotic liver disease (MASLD), cardiovascular disease, and even certain cancers [1,6,7]. In obese people, it is presently accepted that oxidative stress is induced by obesity-related diseases such as hyperglycemia, insulin resistance, diabetes, dyslipidemia, atherosclerosis, and inflammation [1,4,8]. Furthermore, adipose tissue inflammation and oxidative stress cause a dysregulation of adipokine secretion, with a reduction in adiponectin and an increase in the secretion of resistin, leptin, and pro-inflammatory adipokines and cytokines, which contribute to cardiovascular stiffness, impaired vascular relaxation, and finally, cardiac diastolic dysfunction [3]. In addition, the cardiac phenotype in obesity includes concentric left-ventricular hypertrophy (LVH), myocardial fibrosis, microvascular dysfunction, arrhythmia, heart failure (HF)—particularly HF with preserved ejection fraction (HFpEF)—atrial fibrillation (AF), cardiac remodeling, myocardial infarction, and left-ventricular systolic dysfunction, causing deterioration in myocardial function and HF [3,9]. 

**Figure 1:**
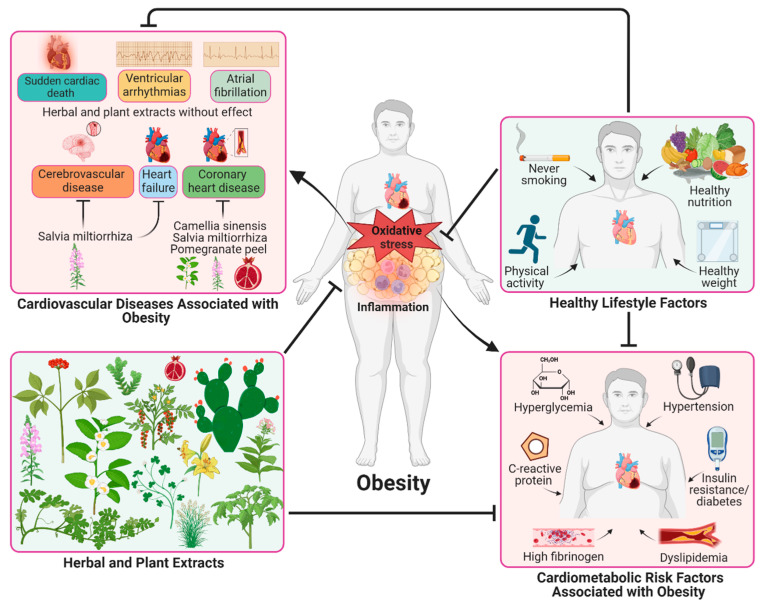
Overview of obesity. Obesity is associated with the development of cardiometabolic risk factors and cardiovascular diseases. However, healthy lifestyle and intake of plant extracts such as those described in this review (21 natural extracts), which have antioxidant and anti-inflammatory properties, can prevent these pathological conditions.

The complex interaction between several cardiometabolic dysfunctions and pathological processes plays an essential role in the pathogenesis of obesity, which is associated with the morbidity and mortality of diabetes and cardiovascular disease [1,4,8,10]. Moreover, cardiovascular diseases are closely associated with high cholesterol, obesity, smoking, diabetes, and a lack of physical activity. Therefore, a convenient lifestyle accompanied by healthy nutrition, a reduction in energy-dense food consumption, physical activity and activities that reduce oxidative stress are the most common therapeutic strategies to reduce cardiometabolic risk factors closely linked to obesity, such as hypertension, dyslipidemia, insulin resistance, diabetes, elevated levels of fibrinogen and C-reactive protein (CRP), including low-grade chronic inflammation (Figure 1) [1,11]. 

Several antiobesity medications approved by the United States (US) Food and Drug Administration (FDA) exist, which include liraglutide (Saxenda), naltrexone-bupropion (Contrave), orlistat (Xenical), phentermine-topiramate (Qsymia), semaglutide (Wegovy), setmelanotide (IMCIVREE), and tirzepatide (Mounjaro) [12,13]; however, the use of these drugs remains controversial, as they are associated with a number of adverse side effects and weight regain when the medication is stopped [12,13]. Therefore, the WHO (Committee, 1980) recommended the use of indigenous medicinal plants to treat obesity because of their easy availability, low costs, and relatively fewer side effects. Moreover, herbs and plants contain an unlimited source of phytochemicals, macronutrients, micronutrients, and antioxidants such as polyphenols, which are known to prevent diseases associated with oxidative stress such as obesity and its related complications.

In this review article, we discussed the beneficial properties of several herbs and plant extracts (21 natural extracts), as well as their active components against obesity, cardiometabolic risk factors, and associated pathophysiological processes to treat and prevent different cardiovascular diseases in preclinical and clinical trials, considering their molecular mechanisms underlying for their medicinal uses.

## 2. Phytochemical Constituents and Pharmacological Activities of Herbs and Plants with Cardiovascular Protective Effects

### 2.1. Allium sativum, Family Alliaceae 

Garlic is one of the most well-known herbal medicines in the world and has been used as a spice or medicinal herb for many centuries. The major bioactive compounds of bulbs include sulfur compounds such as alliin, allicin, ajoene, vinyldithiins, diallyl disulfide, allyl methanethiosulfinate, diallyltrisulfide, dimethylmonotohexasulfide, and S-allylcysteine [14]. Garlic administered either in liquid form or capsules has different antioxidant, antidiabetic, antihypertensive, antiatherosclerotic, anti-inflammation, endothelial-protecting, lipid-lowering, plasma fibrinogen-lowering, platelet aggregation-inhibiting, fibrinolytic activity-increasing, and other cardiovascular-protective effects [14,15,16]. In addition, aged garlic extract (AGE) has been used in previous human trials and has been shown to be safe [17].

### 2.2. Andrographis paniculata (Burm. F.) Wall. Ex Nees (Family: Acanthaceae)

*Andrographis paniculata* (Burm.f.) Nees is considered a potent plant medicinal in most parts of Asia for the treatment of endocrine disorders, inflammation, and hypertension. Based on phytochemical tests, flavonoids, alkaloids, tannins, triterpenoids, and polyphenols have been isolated from *Andrographis paniculata* [18]. In addition, andrographolide is a natural diterpenoid lactone extracted from *Andrographis paniculata* (Burm.f.) Nees, and scientific studies revealed that andrographolide is the main phytoconstituent for its medicinal properties, such as antineoplasm antibacterial, anti-inflammatory, antimalaria, antithrombotic, hepato-protective, antihypertensive, antidiabetic, antioxidant, antiapoptosis, antifibrosis, and cardioprotection activities [19]. 

### 2.3. Aronia melanocarpa (Michx.) Elliott. (Family: Rosaceae)

Black chokeberry, *Aronia melanocarpa* (Michx.) Elliot is a deciduous shrub native to eastern North America, and *Aronia melanocarpa* (chokeberry) contains a rich source of biologically active polyphenols such as anthocyanins, proanthocyanidins, and phenolic acid, which have strong antioxidant effects and cardioprotective benefits [20,21]. Other bioactive compounds have been identified to be present in the fruits and other parts of the plant, such as neochlorogenic and chlorogenic acids, cyanidin-3-galactoside, cyanidin-3-arabinoside, and (-)-epicatechin [21,22]. *Aronia melanocarpa* or black chokeberry has been found in multiple clinical trials to combat hyperglycemia-induced oxidative stress, blood pressure (BP), cholesterol and the macrovascular complications of diabetes, including cardiovascular disease [21,22]. The berries of *Aronia melanocarpa* also possess therapeutic benefits such as gastroprotective, hepatoprotective, antiproliferative, and anti-inflammatory activities [22]. 

### 2.4. Camellia sinensis (Family: Theaceae)

Green tea derived from *Camellia sinensis* leaves is one of the most popular beverages consumed worldwide. The plant is native to East Asia, possibly originating in southern China, including border areas of Myanmar and India [23]. Green tea extract (GTE) contains several bioactive components, including polyphenols, catechins, theobromine, caffeine, and flavonoids. The major catechins in green tea are (-)-epigallocatechin-3-gallate (EGCG), (-)-epigallocatechin (EGC), (-)-epicatechin-3-gallate (ECG), (-)-epicatechin (EC), and (þ)-catechin (C). Among them, EGCG represents approximately 50–70% of the total catechins from green tea leaves and is primarily responsible for the beneficial effect of green tea [23,24]. The polyphenolic compounds in green tea possess antioxidant properties preventing oxidative stress-caused diseases such as cancer, cardiovascular (e.g., stroke, coronary heart disease, and coronary atherosclerosis) and neurodegenerative diseases. In addition, green tea has beneficial effects on cardiovascular risk factors such as hypertension, lipid disorders, diabetes, endothelial dysfunction, and inflammation. Other beneficial effects include antibacterial, antiviral, antimicrobial, antiobesity, antiangiogenic, and antimetabolic syndrome activities [23,24]. 

### 2.5. Caralluma fimbriata (Family: Apocynaceae)

*Caralluma fimbriata*, an edible succulent and wild medicinal plant growing in dry places, is found throughout Asia (Afghanistan, India, Iran, Pakistan, and Sri Lanka), Africa, Arabian Peninsula, Canary Islands, and Southeast Europe. The key phytochemical constituents of the herb are pregnane glycosides, flavone glycosides, megastigmane glycosides, and saponins, including bitter principles, triterpenoids, and other flavonoids [25]. Pregnane glycosides, particularly rich in this plant, are known to suppress hunger and increase endurance. In addition, extracts of *Caralluma fimbriata* have hypoglycaemic, antioxidant, antiadipogenic, antihypertensive properties [25,26,27]. The herb is also used to treat pain, fever, inflammation, and is commonly consumed by ethnic populations of Central India to manage obesity. Bioactive compounds derived from *Caralluma fimbriata* such as flavonoids, saponins, alkaloids, tannins/gallic-tannins, and trigonelline have antioxidant effects. Meanwhile, the compounds flavonoids, saponins, tannins/gallic-tannins, phytosterol, terpenoids, anthraquinones, pregnane glycosides, and trigonelline are responsible for anti-inflammatory properties of *Caralluma fimbriata.* Alkaloids and quercetin found in *Caralluma fimbriata* have antiadipogenic effects. Phytochemicals such as diterpenes, phytosterol, flavonoids, and quercetin have been reported to have antihyperlipidemic effects. Flavonoids modulate blood pressure through the restoration of endothelial function or by affecting nitric oxide levels [25]. 

### 2.6. Cinnamomum zeylanicum (Ceylon cinnamon), Family: Lauraceae

*Ceylon cinnamon* is scientifically known as *Cinnamomum zeylanicum* Blume. Cinnamon is native to Sri Lanka and is one of the most important spices used daily by people around the world. The most important compounds of cinnamon are cinnamaldehyde and trans-cinnamaldehyde, which also are found in the essential oil, and both contribute to fragrance and to the various biological activities observed with cinnamon. In addition, this plant contains a variety of resinous compounds, such as cinnamate, cinnamic acid, and numerous essential oils [28]. Cinnamon has many health benefits, including anti-inflammatory, antioxidant, blood-glucose regulation, insulin sensitivity improvement, antidiabetic, lipid-lowering, antimicrobial, anticancer, and anticardiovascular properties; cinnamon has also been reported to have benefits against neurological disorders, such as Parkinson’s and Alzheimer’s diseases [28,29,30]. 

### 2.7. Citrullus colocynthis (Family: Cucurbitacea)

*Citrullus colocynthis* (L.) Schrad is widely distributed in desert areas around the world, including Sudan, Morocco, Arabian Desert, Jordan, Tunisia, Iran, India, and Pakistan. *Citrullus colocynthis* contains several compounds, mainly cucurbitacins and others such as alkaloids, flavonoids, coumarins, steroids, and phenolic acids [31]. Parts of this plant have been used in traditional medicine, and are widely used to treat constipation, mastitis, joint pain, diabetes, hypertension, inflammation, leukemia, epilepsy, asthma, bronchitis, jaundice, leprosy, rheumatism, common cold, cough, toothache, wounds, and bacterial infection [31,32,33]. Moreover, in diabetic and nondiabetic animal models, aqueous extracts of *Citrullus colocynthis* have hypoglycemic, antidiabetic, hypolipidemic, and antihyperlipidemic effects, including antiplatelet and profibrinolytic activity [31,33,34]. Antioxidant effects of *Citrullus colocynthis* leaf and root extracts have been described; these effects were reported with triterpenoids spinasterol and 22,23-dihydrospinasterol from leaves of *Citrullus colocynthis* [35]. Other compounds, such as their polyphenols, and flavonoids, remove free radicals and thus have a protective effect [33]. Aqueous extracts from roots and stems of the plant and from fruits and seeds displayed anti-inflammatory activities at different doses without inducing acute toxicity [36]. Saponins and catechic tannins from *Citrullus colocynthis* seed extract are bioactive components that contribute to hypolipidemic activity [37]. However, some side effects caused by *Citrullus colocynthis* have been reported, such as nausea, vomiting, colic, diarrhea, hematochezia, and nephrosis [38]. 

### 2.8. Cacao (Theobroma cacao L.), Family: Malvaceae 

*Theobroma cacao* is native to the jungles of South America and then extended to Mexico. Cocoa beans are the seeds, which are used mainly to produce chocolate, cocoa, and fat. Cocoa is one of the richest sources of polyphenols (about 6–8% by dry weight), which include mainly flavonoids, flavanols, flavanones, isoflavones, and nonflavonoids, as well as catechins, anthocyanidins/anthocyanins, flavonol glycosides, and procynanidins [39]. Polyphenols are beneficial on blood pressure, insulin resistance, lipid profile, endothelial dysfunction, and oxidative stress, thus contributing to the prevention of cardiometabolic disorders. Flavanols in cocoa are found as (-)-epicatechin, (+)-catechin, and procyanidins. (-)-epicatechin has the capacity to modulate lipid metabolism (e.g., hypocholesterolemic effect). Black chocolate is considered one of the major sources of antioxidants, and the compounds theobromine, caffeine, (-)-epicatechin, catechins, and oligomeric procyanidins in cocoa have strong antioxidative activities [39,40,41,42]. Dry cocoa powder can contain approximately 200 mg of caffeine per cup, and caffeine progressively reduces body fat mass and body fat in rats fed an HFD [43]. Several therapeutic effects have been attributed to cocoa-derived polyphenols, such as the improvement of lipid peroxidation, insulin resistance, lipid profile, endothelial dysfunction, postprandial systolic blood pressure (SBP), oxidative stress, and inflammation, including lipid metabolism, and glucose metabolism [39,41,42]. Cocoa and its products play an important role in the prevention and treatment of CVDs, including the inhibition of platelet activation and aggregation [44].

### 2.9. Corni Fructus (Cornus officinalis Sieb. et Zucc.), Family: Cornaceae

*Cornus officinalis* Siebold et Zuccarini, usually known as Corni Fructus, is a herb and food plant in East Asia used in traditional Chinese medicine. Several chemical constituents have been identified in Corni Fructus, which are terpenoids, flavonoids, tannins, polysaccharides, phenylpropanoids, sterols, carboxylic acids, furans, saponins, phenolic acid (gallic acid and tannic acid), loganin, and mineral substances. In addition, other phytochemicals are reported in Corni Fructus extracts, such as morroniside, 1,6-α-glucans, loganin, ursolic acid, oleanolic acid, cornuside, polymeric proanthocyanidins, 1,2,3-tri-O-galloyl-beta-D-glucose, 1,2,3,6-tetra-O-galloyl-beta-D-glucose, among others [45,46]. The components in Corni Fructus, such as iridoid glycoside, morroniside, loganin, and polyphenols, exhibit protective effects against hyperglycemia, oxidative stress, and cancer. In vivo and in vitro experimental studies indicate that Corni Fructus has several biological activities, including hypoglycemic, antioxidant, anti-inflammatory, antineoplastic, antimicrobial, anticancer, antiapoptosis, anti-inflammation, antiosteoporosis, immunoregulation, neuroprotective, hepatoprotective, nephroprotective, and cardiovascular protection [45,46,47,48]. However, clinical studies are still needed to confirm the reported pharmacological activities.

On the other hand, Corni Fructus has been frequently used for the treatment of asthenia diseases, liver, and kidney diseases, including reproductive system diseases since ancient times. Moreover, it is commonly used for the treatment of several conditions such as diabetes, frequent urination, impotence, and collapse with profuse sweating [45,46]. 

### 2.10. Cydonia oblonga Miller (Family: Rosaceae)

*Cydonia oblonga* Miller (COM) is a plant known by various names, including quince, aiva, bier, and marmelo. The fruit of COM contains various polyphenolic compounds, organic acids, ionone glycosides, and tetracyclic sesterterpenes, including chlorogenic acid, cryptochlorogenic acid, neochlorogenic acid, isochlorogenic acid, quercetin 3-rutinoside, quercetin 3-galactoside, quercetin 3-glucoside, kaempferol 3-glucoside, kaempferol 3-glycoside, and kaempferol 3-rutinoside. In the pulp, leaves, peel, seeds, and complete fruits of COM, several citric, ascorbic, malic, oxalic, quinic, fumaric, and shikimic acids have been discovered [49,50]. The fruit of COM is commonly used in the Mediterranean region to prevent or treat obesity. In addition, the fruit of COM has been used for the treatment of hypertension, diabetes, cancer, cardiovascular diseases, respiratory disorders, hemolysis, and ulcers [49,51,52,53]. Several studies have reported the beneficial effects of COM extracts, such as antioxidant, anti-inflammatory, antiallergic, antidepressant, and antistress, including positive effects on cardiovascular-associated factors such as BP, glucose metabolism, lipid profile, liver dysfunction, and thrombosis [49,52,53]. Moreover, the plant’s seeds have been used to treat diarrhea, dysentery, constipation, cough, sore throat, and bronchitis [49]. 

### 2.11. Ginkgo biloba (Family: Ginkgoaceae)

For centuries, the herb *Ginkgo biloba* has been used in traditional Chinese medicine to treat various medical conditions. The extracts of *Ginkgo biloba* (EGb) leaves have a wide variety of bioactive compounds, such as flavonoid heterosides (between 22% and 27%), represented by flavonol glycosides (kaempferol, quercetin, myricetin, apigenin, isorhamnetin, luteolin, and tamarixetin), diterpenes, sesquiterpenes, between 5% and 7% of terpene trilactones (ginkgolide A, ginkgolide B, ginkgolide C, ginkgolide J, ginkgolide M, ginkgolide K, ginkgolide L, and bilobalide), 2.8–3.4% corresponding to ginkgolides A, B, and C, and 2.6–3.2% consisting of bilobalide, phenolic acids, polysaccharides, steroids, and a content of less than 5 mg/kg of ginkgolic acids, of which flavonoids and terpene lactones are usually considered to be responsible for the pharmacological activity associated with this plant [54,55]. For instance, ginkgolide B is a potent anti-inflammatory agent and inhibits the platelet-activating factor, and the flavonols of *Ginkgo biloba* have cardioprotective, antioxidant, antibacterial, and neuroprotective properties. Current pharmacological studies have shown that flavonoids from *Ginkgo biloba* have prominent cardioprotective activities, such as regulating blood lipids, lowering blood sugar, inhibiting cardiomyocyte apoptosis, dilating blood vessels, antagonizing platelet-activating factor, and preventing myocardial ischemic injury and vascular rupture [54,55,56,57]. It is important to note that many types of preparations based on *Ginkgo biloba* extract have been developed for the treatment of cardiovascular diseases. *Ginkgo biloba* is also used for the prevention and treatment of hypertension, atherosclerosis, peripheral arterial disease, peripheral venous disease, Raynaud’s phenomenon, and erectile dysfunction. The plant has also been used for diseases such as cognitive decline, dementia, and tinnitus [54,56,58,59]. 

### 2.12. Coffea (Genus Coffea), Family: Rubiaceae

Coffee is widely consumed in the world and has a variety of phytochemicals. The main coffee polyphenols include the glycosylated derivate forms of the polyphenol and chlorogenic acids (CGAs), such as esters of caffeic acid and quinic acid. Green coffee is raw coffee beans that have not been roasted, and it is rich in bioactive phytochemical compounds, mainly CGAs, caffeine, and soluble fiber (mostly galactomannans and arabinogalactan) [60,61]. Green coffee bean extract (GCBE) has antioxidant properties and neutralizes reactive oxygen species. In addition, studies have found that the CGA from GCBE regulates vasoreactivity and glucose metabolism, including properties such as anticancer, anti-inflammatory, antilipidemic, antihypertensive, and antidiabetic effects [62,63,64]. Concerning hypolipidemic effects, GCBE and its CGA reduce triglyceride (TG) and total cholesterol (TC) levels; however, the effects on high- and low-density lipoprotein cholesterol (LDL-C) levels are inconsistent. Some studies have reported an increase in serum high-density lipoprotein cholesterol (HDL-C) after GCBE intake, while others have reported non-significant results [65]. 

### 2.13. Hibiscus sabdariffa (Roselle), Family: Malvaceae 

*Hibiscus sabdariffa* Linn is commonly known as roselle, which probably originated in West Africa and grows in tropical and subtropical regions. Roselle contains several bioactive compounds that have medicinal properties, such as phenolic acids (protocatechuic, chlorogenic caffeic acid, and gallic acids), flavonoids (quercetin-3-glucoside, methyl epigallocatechin, myricetin, quercetin, rutin, and kaempferol), anthocyanins (delphinidin-3-sambubioside and cyanidin-3-sambubioside), and organic acids (hibiscus acid, citric acid, hydroxycitric acid, malic acid, and tartaric acid), which are responsible for many biological activities [66,67]. This plant is commonly used as a traditional drink material and folk medicine against hypertension, pyrexia, liver disease, fever, inflammation, kidney and urinary bladder stones, and obesity. Roselle, mainly its calyx, has phytochemicals with various health benefits, such as antihyperglycemic, antihyperlipidemic, antihypertensive, antioxidative, anti-inflammatory, and antifibrosis effects [66,67,68,69]. Roselle water extracts also show anticancer, antibacterial, nephro- and hepato-protective, renal/diuretic effect, anticholesterol, and antidiabetic effects among others; this might be related to the inhibition of α-glucosidase and α-amylase, inhibition of angiotensin-converting enzymes (ACE), including the direct vasorelaxant effect or calcium channel modulation [66,67,70,71]. Additionally, *Hibiscus sabdariffa* relaxes other smooth muscles, including the intestine, uterus, and bladder [67]. 

### 2.14. Ilex paraguariensis A.St.-Hil. (Mate), Family: Aquifoliaceae

*Ilex paraguariensis*, commonly known as yerba mate, is one of the most widely consumed plants in subtropical regions of South America (Brazil, Paraguay, Uruguay, and Argentina). This tree or shrub contains polyphenols derived from caffeoyl, manly monocaffeoyl quinic isomers (3-O-caffeoyl quinic or neochlorogenic acid, 5-O-caffeoyl quinic or chlorogenic acid and 4-O-caffeoyl quinic or cryptochlorogenic acid), caffeic acid, and dicaffeoyl quinic isomers (3,4-dicaffeoylquinic acid, 3,5-dicaffeoylquinic acid, and 4,5-dicaffeoylquinic acid), methylxanthines (caffeine, theophylline, and theobromine), flavonoids (quercetin, kaempferol, and rutin), tannins, and numerous triterpenic saponins that are derived from ursolic acid and are named metesaponins [72,73]. Yerba mate exhibits various biological activities such as antioxidant, anti-inflammatory, antiobesity, anticancer, immunomodulatory, improvement in glycemic and lipid metabolism, reversion of insulin resistance, inhibition of glycation and atherosclerosis, thermogenic and vasodilatation effects, a protective effect against induced DNA damage, and reduction in cardiovascular risk [72,73,74,75,76,77]. Moreover, yerba mate facilitates recovery from physical and mental fatigue, reduces the feeling of hunger, and works as a diuretic; an aqueous extract of this medicinal plant protects the myocardium against ischemia-reperfusion injury and decreases oxidative damage, which can be attributed to the potent antioxidant properties of the extract [72,78]. 

### 2.15. Moringa oleifera Lam., Family: Moringaceae

*Moringa oleifera* Lam. is native to the sub-Himalayan northern parts of India and commonly cultivated throughout tropical and sub-tropical countries. *Moringa* leaves are rich in many nutritious and bioactive compounds, including carotenoids, polyphenols, glucosinolates [the most abundant of them is 4-O-(α-l-rhamnopyranosyl-oxy)-benzylglucosinolate or also named glucomoringin], tannins, among others. Polyphenolic compounds are represented by flavonoids (mostly quercetin and kaempferol in their 3′-O-glycoside forms) and phenolic acids such as gallic, chlorogenic, which is an ester of dihydrocinnamic acid (caffeic acid), ellagic, quinic, and ferulic acids [79,80]. The following compounds have hypotensive properties, such as sothiocyanates, thiocyanates, and nitriles, which are formed by enzymatic hydrolysis of the glucosinolates; niaziminin, also a hypotensive, is a mustard oil glycoside isolated along with other glycosides (niazinin and niazimicin) from ethanolic extracts of *Moringa oleifera* leaves. The flavonol quercetin is a potent antioxidant and is found at concentrations as high as 100 mg/100 g of dried *Moringa oleifera* leaves [79,80]. The bioactive compounds of *Moringa oleifera* are accountable for many medicinal properties such as cholesterol-lowering, antiobesity, antihyperlipidemic, antidiabetic, antihypertensive, neuroprotective, antiasthmatic, antitumor, anti-inflammatory, antioxidant, antipyretic, antiepileptic, antiulcer, antispasmodic, diuretic, hepatoprotective, antiviral, antimicrobial, antifungal, and cardioprotective activity, as well as protection against signs of aging, typhoid fever, malaria, diarrhea, and dysentery [79,80,81,82,83,84]. 

### 2.16. Nigella sativa, Family: Ranunculaceae

This medicinal plant is popularly known as black seed or black cumin and is mainly distributed in North Africa, the Middle East, Europe, and Asia. The major phytochemical constituent of the seeds from *Nigella sativa* is thymoquinone (particularly the essential oil), but they also include phytosterols (β-sitosterol and stigmasterol), alkaloids (e.g., nigellamines), saponins, dithymoquinone, nigellin, terpenes and terpenoids (such as thymoquinone and its derivatives), tocopherols, polyphenols (such as quercitrin and kaempferol), and miscellaneous components [85,86]. These bioactive components of the seeds are responsible for the pleiotropic pharmacological properties, such as antioxidant, anti-inflammatory, antihypertensive, antihepatotoxic, anticancer, hypoglycemic, antimicrobial, antifungal, antinephrotoxic, antihepatotoxic, lipid-lowering properties, and immunostimulating activities. The seeds of *Nigella sativa* are also used for the treatment of cardiovascular diseases, respiratory diseases (asthma and bronchitis), cough, headache, rheumatic disorders, fever, influenza, obesity, epilepsy, back pain, and gastrointestinal disorders (indigestion and diarrhea) as well as in cases of amenorrhea, dysmenorrhea, and skin infections [85,86,87]. 

### 2.17. Opuntia ficus Indica, Family: Cactaceae

The species of genus *Opuntia* (approximately 200) grow extensively in desert or semi-desert regions in Mexico, the United States, and Mediterranean countries, among other countries. This plant is native to Mexico and is known there as nopal, prickly-pear cactus in the Southern United States, and Indian fig cactus in Europe. Cladodes of *Opuntia ficus-indica* provide dietary fiber and bioactive compounds such as carotenoids (lutein, β-carotene, and β-cryptoxanthin), flavonoids (isorhamnetin-3-O-glucoside, kaempferol, quercetin, isoquercetin, nicotiflorin, and rutin), and phenolic compounds (coumaric callic acid, and 3,4-dihydroxybenzoic 4-hydroxybenzoic, and ferulic acid). Moreover, cladodes are rich in pectin, mucilage, minerals, malic acid, vitamins, and antioxidants. Meanwhile, prickly-pear fruits contain bioactive compounds such as pigments (betaxanthins, betacyanins, and betalains) and flavonoids (kaempferol, quercetin, and isorhamnetin) [88,89]. *Opuntia ficus-indica* has actions against atherosclerotic cardiovascular diseases, diabetes, obesity, hypertension, asthma, burns, edema, and indigestion, as well as other pharmacological effects including antioxidant, neuroprotective, anti-inflammatory, antihypercholesterolemic, antiulcer, antimicrobial, antiviral potential, wound-healing, skin-protective, hepatoprotective, anticancer, human infertility, and chemopreventive effects. Moreover, *Opuntia ficus*-*indica* has effects on the bone health, kidneys, and gastrointestinal tract, including gastroprotective, sedative, analgesic, anxiolytic, cognitive, and memory effects [88,89,90,91,92]. 

### 2.18. Platycodon grandiflorus, Family: Campanulaceae

*Platycodon grandiflorus*, a common Chinese herb, is mainly distributed in Northeast Asia, including China, the Korean Peninsula, Japan, and Siberia, where it has been used for decades as a traditional medicinal herb. A phytochemical investigation revealed that *Platycodon grandiflorus* contains at least 100 compounds, including steroidal saponins, flavonoids, polyacetylenes, sterols (e.g., stigmasterol), phenolic acids, and other bioactive compounds, among which, saponins are considered the main active compounds [93,94]. Triterpenoid saponins such as platycodin A, C, D and polygalacin D, which contain sterol components including stigmasterol, have various effects such as improving blood glucose and cholesterol metabolism, anticancer and anti-inflammatory effects, and relieving atopic dermatitis. Platycodin A, platycodin C, deapioplatycodin D, and 16-oxo-platycodin D have antiobesity effects. Platycodin D is the main phytochemical in *Platycodon grandiflorus* extract, which has shown antioxidant, anti-inflammation, antiadipogeneic, antiobesity, antifibrosis, and antitumor properties. Furthermore, platycodin D inhibits pancreatic lipase activity [93,94] and inhibits the adipogenesis of 3T3-L1 cells by upregulating Kruppel like factor 2 (KLF2) and subsequent downregulation of peroxisome proliferator-activated receptor gamma (PPARγ) [95]. Platycodin D improves obesity in db/db mice by an AMPK-associated decrease in adipogenic (PPARγ and CCAAT/enhancer-binding protein alpha, C/EBPα) and increase in thermogenesis markers (UCP1 and PGC1α) [96]. Moreover, total saponins of *Platycodon grandiflorus* reduce the levels of blood glucose, serum cholesterol, TG, and LDL-C, increase serum HDL-C, and improve liver function, thereby reducing T2D in rats [94]. In summary, *Platycodon grandiflorus* exhibit pharmacological activities, such as antidiabetic, antibacterial, antiapoptosis, hypocholesterolemic, hypoglycemic, immune enhancement, and liver protection effects, improve insulin resistance and the lipid profile, decrease BP, alleviate atopic dermatitis, as well as relieving cough and asthma activities, apophlegmatic, antitussive, and cardiovascular system activities [93,94,97,98,99,100]. *Platycodon grandiflorus* has also been reported to be used for the treatments of chest congestion, chest distress, diphtheria, dyspnea, mastitis, measles, dermatitis, dysentery, suppuration, chronic rhinitis, chronic tonsillitis, bronchitis, asthma, pulmonary abscesses, pulmonary tuberculosis, faucitis, bronchial asthma, and other conditions [93,94]. 

### 2.19. Punica granatum L., Family: Lythraceae

*Punica granatum* Linn., commonly known as pomegranate, is a small shrub with tasty fruit native to the Middle East, growing in subtropical and temperate regions and having a variety of planting distributions around the world. More than 60 bioactive components have been identified in pomegranate, which are categorized as phenols, flavonoids, triterpenes, alkaloids, sterols, vitamins, and unsaturated fatty acids. In addition, pomegranates are rich in polyphenolic antioxidants, such as tannins, anthocyanin, and flavonoids; these active components are the most abundant in pomegranates. The main compounds isolated from pomegranate flowers are polyphenols, flavonoids, terpenoids, and triterpenoids, such as leanolic acid and ursolic acid. The pomegranate fruit includes hydrolyzable tannins like gallotannins and ellagitannins, as well as ellagic acid and its derivatives, gallic acid, anthocyanins, proanthocyanidins, flavonoids, sterols, lignans, terpenes, and terpenoids. Pomegranate peel is abundant in a variety of phenolics, ellagitannins, proanthocyanidins, microelements, and flavonoids, including kaempferol-3-O-glucoside. *Punica granatum* bark is rich in tannins, proanthocyanidins, anthocyanins, and terpenoids. Pomegranate juice is rich in antioxidants, such as polyphenols, flavonoids, ellagitannins, tannins, and anthocyanins [101,102,103]. In vivo and in vitro studies have shown that extracts of different pomegranate fractions (peels, flowers, seeds, and juice) improve lipid metabolism in diseases such as atherosclerosis, metabolic dysfunction-associated steatotic liver disease (MASLD), metabolic syndrome, and type 2 diabetes, including a wide range of diseases, such as inflammation, Alzheimer’s disease, ulcers, diarrhea, erectile dysfunction, obesity, cancer, brain ischaemia, fibrosis, and fungal and microbial infections. Pomegranate flowers are used for the treatment of cardiovascular disorders, diabetes, obesity, and some microbial infections (Salmonella entteriditis and Kentucky). Pomegranate seeds are used to treat heart diseases, diabetes, cancer, obesity, urinary disorders, and to prevent miscarriage and to improve male fertility. Additionally, pomegranate seeds have antimicrobial and antioxidant properties. Pomegranate peel extracts are traditionally used to treat diarrhea and ulcers. Other pharmaceutical properties reported in pomegranate peels include antiproliferative, anti-inflammatory, antioxidant, and anticancerous effects. *Punica granatum* bark has been used traditionally for the treatment of inflammation, diarrhea, malaria, nose bleeding, sore throat, ulcer, and hoarseness. Pomegranate juice has important biological actions, including antioxidant activity and cardiovascular protection. Moreover, the consumption of pomegranate can relieve dental infections and menopausal symptoms, as well as improve the intestinal microbiota, thus preventing obesity and diabetes [101,102,103,104,105,106]. 

### 2.20. Salvia miltiorrhiza Bunge, Family: Lamiaceae 

*Salvia miltiorrhiza* Bunge, commonly called danshen, is a perennial herb used in traditional Chinese medicine. *Salvia miltiorrhiza* contains more than 100 compounds, including salvianolic acid A/B/C/D/E/F/G, lithospermic acid, danshensu, caffeic acid, and rosmarinic acid, tanshinone I/IIA/IIB/V/VI, tanshindiol A, cryptotanshinone, dihydrotanshinone I, miltirone, dehydro miltirone, and isotanshinone, among others. The bioactive compounds in *Salvia miltiorrhiza* extract are classified into two major groups, water-soluble phenolics (salvianolic acid and comfrey acid) and liposoluble tanshinones (diterpenoids), which are responsible for the main pharmacological properties of *Salvia miltiorrhiza* [107,108]. Tanshinone IIA and salvianolate have various cardiovascular and pharmacological effects, including antioxidative, anti-inflammatory, endothelial protective, anticoagulation, vasodilation, myocardial protective, anticoagulation, vasodilation, and antiatherosclerosis, as well as effects on reducing the proliferation and migration of vascular smooth muscle cells. Additionally, salvianolates are composed of salvianolic acid B, rosmarinic acid, and lithospermic acid, which are widely used in the treatment of coronary heart disease. Meanwhile, tanshinones are more effective in the treatment of cardiovascular diseases and cerebrovascular diseases, including atherosclerosis, myocardial infarction, cardiac hypertrophy, myocardial ischemia-reperfusion (I/R), and chronic heart failure. *Salvia miltiorrhiza* has other effects such as antidiabetic, anti-inflammation, antioxidant, antifibrosis, and antiapoptosis effects. *Salvia miltiorrhiza* is also used to treat malignant tumors, neurological, lung diseases, inflammatory diseases, gynecological diseases, liver diseases, renal diseases, and metabolic disorders such as atherosclerosis, hyperlipidemia, obesity, and other dyslipidemia-related diseases [107,108,109,110]. 

### 2.21. Taraxacum officinale L. (Dandelion), Family: Asteraceae

*Taraxacum officinale* L., also known as dandelion, a perennial herb and commonly regarded as a weed, is native to Eurasia and grows in America, Africa, New Zealand, and Australia. Dandelion has phenolic acids (chlorogenic acid and chicoric acid), flavonoids (luteolin derivatives and quercetin), and terpenes (sesquiterpene lactones). The leaves contain bitter sesquiterpene lactones (taraxinic acid and triterpenoids such as cycloartenol), while the roots have phenolic acids, inulin, sesquiterpene lactones, triterpenes, sterols (taraxasterol, taraxerol, cycloartenol, beta-sitosterol, and stigmasterol), and the compounds already mentioned, which contribute to their therapeutic properties [111,112]. Dandelion has been used as a phytomedicine for its holeretic, antirheumatic, diuretic, antibacterial, hypolipidemic, hypoglycemic, antithrombotic, anti-inflammatory, antiobesity, antioxidant, and antiplatelet effects, as well its use against cancer and cardiovascular ailments. Moreover, dandelion is used as a remedy for kidney diseases, and liver, kidney, and spleen disorders. Dandelion has high levels of phenolic acids, with antioxidant effects; coumarins with anti-inflammatory, anticancer, antibacterial, and antithrombotic properties; sesquiterpene lactones with anti-inflammatory and antibacterial effects; and triterpenes or phytosterols, which possess antiatherosclerotic effects. Dandelion leaves and flowers contain polyphenols, predominantly hydroxycinnamic acid derivatives, and flavonoids (apigenin and luteolin derivatives), all of which have antioxidant and hypocholesterolemic properties. Dandelion roots are rich in inulin, which has a hypoglycemic, probiotic, and immune-boosting effect; meanwhile, its phytochemicals such as phenolic acids and sesquiterpene lactones are responsible for its antidiabetic properties. In general, bioactive compounds from dandelion roots possess bifidogenic, anti-inflammatory, and antifibrotic activities [111,112,113,114,115,116].

## 3. Pathological Processes Involved in Obesity

Overweight and obesity are increasingly common conditions in the world due to the intake of calorie-dense foods and relatively inactive lifestyles, which create long-term imbalances between energy uptake and expenditure, and these conditions promote the deposition of fat mass in the body’s WAT, leading to phenotypic changes in this tissue such as adipocyte hypertrophy (cell size increase) and subsequently hyperplasia (cell number increase) [1,3,117]. In obesity, the hypertrophied WAT visceral adipocytes show lipolysis activation, leading to high levels of circulating non-esterified fatty acids (NEFAs) [118]. NEFAs in normal conditions are catabolized by the β-oxidation to provide energy to tissues such as the liver and muscle; however, high concentrations contribute to the development of insulin resistance [119]. Furthermore, hypertrophic visceral adipocytes contribute to elevated circulating triacylglycerol (TAG) levels mainly from de novo lipogenesis, in which fatty acids (FAs) are synthesized from carbohydrates or FAs are provided from chylomicrons and very low-density lipoproteins (VLDL) [120]. Several studies suggest that oxidative stress plays a fundamental role as a factor linking obesity and its related complications. Furthermore, oxidative stress can increase with preadipocyte proliferation, adipocyte differentiation, and the size of mature adipocytes. Nuclear factor erythroid 2-related factor 2 (NRF2) is a key transcription factor that protects against oxidative stress and electrophilic stress. NRF2 is abundantly expressed in WAT. The deficiency of NRF2 leads to imparting adipocyte differentiation in 3T3-L1 cells and human subcutaneous preadipocytes. Meanwhile, its transfection stimulates hormone-induced adipocyte differentiation. Oxidative stress is increased in WAT from obese mice and from human individuals with obesity. NRF2 in response to oxidative stress activates *Srebf1* promoter and induces target gene transcription and subsequent lipogenesis, which promotes lipid accumulation in adipocytes, thus exacerbating the development of obesity [121]. However, the effects of NRF2 are controversial and even contradictory in animal models of diet-induced obesity. mRNA expression and protein levels of *Nrf2* gene were reduced or increased in WAT and liver in obese mouse models with different percentages of fat calories and feeding duration [122]. Obesity per se can also induce systemic oxidative stress through superoxide generation from NADPH oxidases, oxidative phosphorylation, protein kinase C activation (PKC), glyceraldehyde auto-oxidation, and polyol and hexosamine pathways. Additionally, elevated plasma-free FAs promote the generation of superoxide radicals, hyperleptinemia, low antioxidant defense, and low-grade chronic inflammation. Additionally, postprandial reactive oxygen species generation are other factors that also contribute to oxidative stress in obesity. Obesity-associated oxidative stress induces various pathological events, including insulin resistance and diabetes, liver failure, cardiovascular complications, sleep disorders, and asthma, including reproductive, oncological, and rheumatological problems [1,3,123]. Adipose tissue produces several adipokines including cytokines and hormones, which regulate energy homeostasis, glucose and lipid metabolism, and cardiovascular functions [124]. Because obesity is directly associated with low-grade chronic inflammation, the hypertrophy and hyperplasia of adipose tissue leads to the organ’s dysfunction and development of a pro-inflammatory microenvironment, and the signaling pathway of NF-κB is activated and increases the expression of interleukin-6 (IL-6), tumor necrosis factor-α (TNF-α), and IL-1β through the TLR4/MyD88 signaling pathway; other molecules increased with excessive adiposity are leptin, IL-8, CRP, PAI-1, haptoglobin, angiotensinogen, inducible nitric oxide synthase, platelet-activating factor (PAF) and chemokines, such as monocyte chemotactic protein 1 (MCP1), which promotes the migration of macrophages into the adipose tissue and induces the release of cytokines. Furthermore, in obese subjects, the levels of interleukin 10 (IL-10) are decreased, which worsens the metabolic profile due to IL-10 inhibiting the synthesis of pro-inflammatory cytokines [3,19,123,125,126]. The activation of NF-κB, TLR4, and mTOR leads to the attenuation of insulin signaling and insulin resistance in several tissues, which contribute to obesity-related complications, including diabetes and atherosclerosis [127,128]. Platelets are key players in thrombotic processes, and various platelet markers have been reported elevated in obese and T2D individuals, including the mean platelet volume, circulating levels of platelet microparticles, oxidation products, platelet-derived soluble P-selectin, and CD40L. Therefore, these markers contribute to an intersection between obesity, inflammation, and thrombotic phenotype [129]. Obesity is an altered health condition with changes in gut microbiota due to the consumption of improper diet, which affects the health status of the host. The gut microbiota plays an important role in energy balance, intestinal integrity, and immunity against invading pathogens. Approximately 100 trillion microbes colonize the human gut, which are represented by microorganisms such as bacteria, archaea, fungi, protozoa, and viruses. The gut microbiota is represented by the phyla Bacteroidetes, Firmicutes, Proteobacteria, Actinobacteria, and Verrucomicrobia, and approximately 90% of the total bacterial species belong to Bacteroidetes and Firmicutes. Obese individuals exhibit reduced proportions of Bacteroidetes and elevated levels of Actinobacteria with no significant differences in Firmicutes; thus, an increased Firmicutes/Bacteroidetes ratio is a biomarker of obesity susceptibility [130,131]. 

### Plant and Herb Extracts with Antiobesity Activity 

Previous studies have shown the nutritional and beneficial effects on the metabolism of plant and herb consumption, showing a wide range of bioactive compounds, many of them with different properties for healthy humans. There are several papers in which the use of various medicinal plants and herbs to treat obesity has been investigated. For instance, green tea is associated with beneficial health effects due to its body-fat-reducing and hypocholesterolemic properties [132,133], and green tea aqueous extract (GTAE, 1.1% and 2%) administered in rats fed with a high-fat diet, lowed atherogenic index, reduced body weight gain (only with 2% of GTAE), and prevented visceral fat accumulation [134]. The visceral weight loss and improvement of lipid profile with green tea in rats may be due to increased thermogenesis and fat oxidation [135]. The main phytochemicals in *Caralluma fimbriata* are pregnane glycosides, which are known to suppress hunger and increase endurance. Therefore, pregnane glycosides and diterpenes from *Caralluma fimbriata*, have antiobesity activities [25]. In a diet-induced obesity (DIO) rat model, *Caralluma fimbriata* extract (CFE) showed dose-dependent appetite suppressant, prevented liver weight and fat pad mass, and protected against atherogenesis in rats fed a cafeteria diet [136]. In overweight adults, CFE preserved body weight, decreased waist circumference, and reduced daily caloric intake over a 16-week period in overweight patients compared to a placebo. The mechanism for appetite suppression by CFE includes a reduction in ghrelin synthesis in the stomach and neuropeptide Y in the hypothalamus [26]. Bioactive compounds derived from *cacao*, which are rich in polyphenols (flavonoids), are beneficial against overweight and lipid disorders. In an obesity model induced by a high-fat diet (HFD) and fructose ingestion in rats, cocoa extracts made with outer pod husk and kernel husk decreased weight gain, reduced SBP, and improved lipid profiles [42]. *Ginkgo biloba* extract (GBE), which contains a mixture of polyphenols with antioxidant properties, has several potentially beneficial effects, such as reducing food and energy intake, reducing body adiposity, improving insulin sensitivity, enhancing insulin receptor and AKT phosphorylation, reducing NF-κB p65 phosphorylation in retroperitoneal adipose tissue of obese rats, and reducing weight gain in models of obesity induced by diet and ovariectomy [137,138,139]. These findings were confirmed in HFD-induced obese male rats; GBE supplementation reduced energy intake and epididymal adipocyte volume [140]. Green coffee beans contain phytochemicals with beneficial effects on cardiometabolic disorders. The 3-caffeoylquinic acid (3-CQA) in green coffee bean extract (GCBE) was evaluated in HFD-induced obese mice, and this extract decreased body weight gain, liver weight and WAT weights, regulating adipogenesis and lipid metabolism-linked genes and proteins in WAT and liver [141]. In addition, in male albino Wistar rats fed with a HFD, and intervened with GCBE when obesity was established, the extract decreased the body and organ weights, and reduced TC, TG, LDL-C, VLDL, glucose, and insulin levels. GCBE also exhibited an increase in adiponectin levels and decreased the expression of RBP4, whereas an increase in GLUT4 expression was observed in the adipose tissue [142]. *Moringa oleifera* leaf extracts (MOLE) possess antiobesity effects in experimental animal models and were tested in HFD-induced obesity in rats. Thirteen metabolites were identified in MOLE, including flavanols, flavones, and phenolic acid. MOLE reduced weight gain and adiposity index, including glucose, insulin and HOMA-IR, and Revised Quantitative Insulin Sensitivity Check Index (R-QUICKI) was significantly increased by MOLE. In visceral fat mass, MOLE significantly reduced the levels of leptin and vaspin; meanwhile, adiponectin, omentin and glucose transporter 4 (GLUT4) expression were increased. MOLE significantly inhibited FAS and HMG-CoA reductase and elevated the mRNA expression of MC4R and PPARα. In obese patients, the administration of *Moringa oleifera* hard gelatin capsules showed a significant reduction in the average BMI, TC and LDL-C [81]. Previous studies have shown that *Ilex paraguariensis* (yerba mate) aqueous extracts inhibited the progression of atherosclerosis and decreased body weight, visceral fat, serum lipids, glucose, leptin, and insulin levels in HFD-fed-rats [143,144]. Treatment with *Ilex paraguariensis* extracts (IPE) in C57BL/6J mice fed a HFD also reduced the accumulation of lipids in adipocytes, body weight gain, and obesity. Additionally, the extract reduced serum cholesterol, serum TG, and glucose levels [76]. These findings were confirmed in another study, in which Yerba mate extract was also evaluated in mice fed with a HFD, and obese mice treated with yerba mate exhibited marked attenuation of weight gain, adiposity, a reduction in epididymal fat pad weight, and restoration of the serum levels of cholesterol, TGs, LDL-C, and glucose [75]. In obese rats primed by early weaning, IPE reversed abdominal obesity, leptin resistance and hypertriglyceridemia [145]. Chlorogenic acid in yerba mate is mainly responsible for these effects [146]. In another study *Ilex paraguariensis* was investigated in Korean subjects with obesity. Individuals with obesity were given oral supplements of Yerba Mate capsules, and this supplementation decreased body fat mass, percentage body fat, and waist–hip ratio, which suggests that Yerba Mate supplementation may be an alternative for treating obese patients [147]. *Platycodon grandiflorus* root extract (PGE) was analyzed in obese Korean adults and revealed a significant decrease in body fat mass and body fat percentage, suggesting antiobesogenic effects in overweight or obese adult humans [148]. Pomegranate extract (PomE) is rich in punicalagins and increases markers related to browning and thermogenesis in human differentiated adipocytes [149]. In addition, in a preclinical study of HFD-induced obesity, PomE increased systemic energy expenditure, thus contributing to a reduction in the low grade of chronic inflammation and insulin resistance associated with obesity [104]. 

Oxidative stress results from the elevated production of free radicals along with reduced levels of antioxidants and plays an important role in cardiovascular diseases, including atherosclerosis and coronary artery disease [123]. CFE improves HFD-induced cardiac damage through reducing cardiac lipids such as total lipids, TG, TC, and free fatty acids (FFAs). Furthermore, CFE improves the activities of antioxidant enzymes, such as glutathione peroxidase (GPx), glutathione reductase (GR), glutathione peroxidase (GPx), catalase (CAT), superoxide dismutase (SOD), and glutathione-s-transferase (GST) [27]. *Moringa oleifera* leaf extracts (MOLE) were evaluated in HFD-induced obesity and cardiac damage in rats. The activities of antioxidant enzymes were decreased in animals that received an HFD; however, these antioxidant enzymes were significantly, and dose-dependently, enhanced by administration with MOLE [150]. 

Several diseases associated with obesity, such as dyslipidemia, T2D, and cardiovascular disease, are closely related to low-grade inflammation. In WAT from obese mice, TNF-α, IL-6, leptin, CCR2, CCL2, and PAI-1 genes were upregulated. However, yerba mate extract administration decreased the expression of these genes [75]. In addition, yerba mate extract intake blunted the proinflammatory effects of HFD-induced obesity in rats through the phosphorylation of hypothalamic IKK and NF-κB p65 expression and increasing the protein levels of IκBα, adiponectin receptor-1, and IRS-2 [151]. *Andrographis paniculata* extract (APE) and its bioactive constituent andrographolide are known to possess anti-inflammatory and antiapoptotic effects. APE was analyzed in myocardial tissue from HFD-induced obese mice. The animals fed with HFD developed myocardial inflammation, which potentially contributed to cardiac hypertrophy and myocardial apoptosis, but APE showed significant inhibition of these effects in obese mice [152]. 

*Aronia melanocarpa* contains a high content of procyanidins and anthocyanins. It was found that *Aronia melanocarpa* extract significantly inhibits the amidolytic activity of thrombin and plasmin, the latter being the main fibrinolytic enzyme [153]. Moreover, patients with metabolic syndrome showed a significant reduction in the levels of TC, LDL-C, and TG, as well as an improvement in platelet aggregation, clotting, and fibrinolysis after *Aronia melanocarpa* extract supplementation [154]. The effect of *Citrullus colocynthis* was investigated on blood hemostasis in HFD-induced obese rats, and it was found that *Citrullus colocynthis* reversed HFD-induced increases in fibrinogen and von Willebrand factor; thus, *Citrullus colocynthis* has antiplatelets and profibrinolytic properties due to its potent hypoglycaemic and hypolipidaemic effect, its ability to reduced levels of circulatory TNF-α and IL-6, and its ability to lower prothrombotic leptin levels and elevate antithrombic adiponectin levels [34]. *Cydonia oblonga* is traditionally used in Uyghur medicine to prevent cardiovascular diseases. *Cydonia oblonga* extract (COE) was explored on models (mice and rats) and markers of thrombosis. COE dose-dependently prolonged bleeding and the clotting time. In addition, COE reduced pulmonary embolism mortality, dose-dependently increased thrombolysis, and reduced TXB2. Therefore, COE has an antithrombotic effect, probably at least in part related to antithromboxane activity [53]. Garlic also inhibits platelet aggregation, and aged garlic extract (AGE) blocks both the activation and aggregation of human platelets. The mechanism implicated by AGEs in the inhibition of platelet aggregation includes an increase in cAMP levels through inhibition of cAMP phosphodiesterase activity, resulting in reduction in calcium mobilization and, therefore, suppresses the binding of GPIIa/IIIb receptors to fibrinogen [155]. GBE was investigated on experimental cardiac remodeling in rats induced by acute myocardial infarction. The results suggest that GBE may inhibit experimental myocardial remodeling in rats after acute myocardial infarction by reducing the transcription of TGF-β1, MMP-2 and MMP-9 genes and attenuating extracellular matrix deposition by decreasing the levels of proteins such as type I collagen, MMP-2, and MMP-9 [156]. 

**Figure 2:**
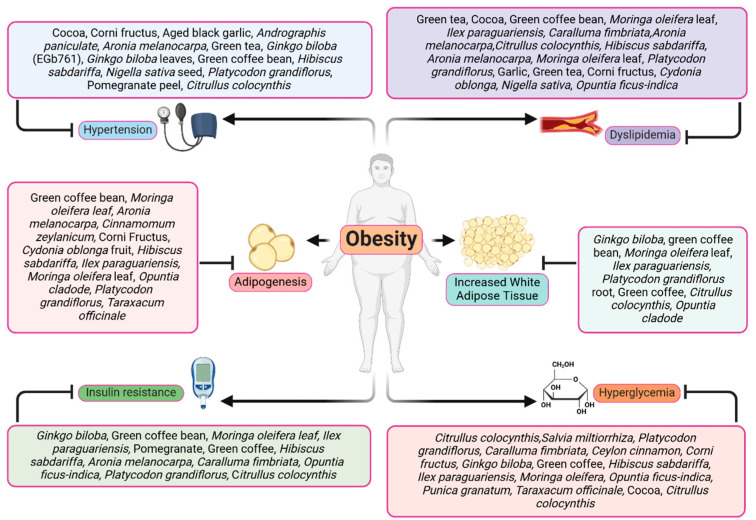
Herb and plant extracts with inhibitory effects on adipogenesis, white adipose tissue accumulation, and cardiometabolic risk factors associated with obesity.

Cardiometabolic parameters were evaluated in ApoE^−/−^ mice fed an atherogenic diet and green coffee extract (GCE). Although GCE did not decrease atherosclerotic lesion progression or plasma lipid levels, it improved metabolic parameters, such as fasting glucose, insulin resistance, serum leptin, urinary catecholamines, and liver TGs. GCE also decreased weight gain, reduced adiposity, lowered inflammatory infiltrate in adipose tissue, and protection against hepatic damage. Furthermore, the number of observed operational taxonomic units (alpha diversity) diminished in ApoE^−/−^ mice with an atherogenic diet, and it was recovered in the GCE-treated ApoE^−/−^ mice [157]. *Hibiscus sabdariffa* extract (HSE) was evaluated in an experimental model of HFD-induced obesity in mice. HSE reduced weight in mice fed an HFD and improved glucose tolerance, insulin sensitivity and normalized LDL-C/HDL-C cholesterol ratio. HSE reduced the expression of different adipokines and pro-inflammatory mediators, and reinforced gut integrity by reducing the Firmicutes/Bacteroidetes ratio [158]. *Salvia miltiorrhiza* extract (SME) was investigated in rats with HFD-induced obesity. SME treatment markedly reduced weight, body fat index, lipid profile, glucose levels, and adipocyte vacuolation. The beneficial effects were accompanied by elevated concentrations of lipid factors such as cAMP, PKA, and HSL in the liver and adipose tissues, enhanced gut integrity, and ameliorated lipid metabolism. Furthermore, *Salvia miltiorrhiza* extract reversed HFD-induced dysbacteriosis by promoting the abundance of Actinobacteriota and Proteobacteria and reducing the growth of Firmicutes and Desulfobacterita [110]. Therefore, the consumption of products derived from the plants and herbs previously analyzed should be considered a new therapeutic strategy in the control of obesity and its associated disorders (Table 1 and Figure 2).

## 4. Adipogenesis and Obesity

Accumulation and adipocyte differentiation are linked with the development of obesity. In the process of preadipocyte to adipocyte differentiation, several transcription factors participate, the most important being cAMP response element-binding protein (CREB), C/EBPα C/EBPβ, C/EBPδ, and PPARγ, which control adipocyte differentiation. Adipocyte differentiation starts with CREB phosphorylation by PKA and ERK1/ERK2, and at the same time, the activation of C/EBPβ and C/EBPδ occurs, which in turn activate C/EBPα and PPARγ [159]. However, this process is much more complicated because it involves other biological signaling pathways. In addition to adipocyte differentiation, PPARγ plays an important role in lipid storage and glucose homeostasis and is predominantly expressed in adipose tissue [160]. On the other hand, the PI3K/AKT pathway plays a critical role in transmitting insulin action in adipose tissue (increases glucose uptake by the GLUT4 membrane translocation) during the adipogenesis of both WAT and brown adipose tissue (BAT). AKT is essential in inducing PPARγ expression. The activation of PI3K/AKT signaling determines the initiation of adipogenic transformation and adipocyte hyperplasia [161]. AMPK is a serine/threonine kinase that is expressed in several tissues (adipose, skeletal, liver, kidney, and hypothalamus), which regulates lipid/glucose homeostasis, autophagy, mitochondrial biogenesis, protein homeostasis, redox equilibrium, food intake, and insulin signaling. AMPK has a function as a cellular energy sensor. AMPK and adiponectin act in peripheral tissues and the central nervous system by regulating food intake. Consequently, the inhibition of hypothalamic AMPK activity along with an increase in adiponectin levels reduces food intake. In addition, AMPK inhibits de novo synthesis of cholesterol, FAs, and TGs, and activates FAs uptake and β-oxidation. It inhibits and phosphorylates proteins involved in the synthesis of FAs (FAS, ACC1, and SREBP-1c). AMPK inhibits the synthesis of cholesterol (phosphorylates and inhibits HMG-CoA reductase) and, through PGC-1α activation, stimulates mitochondrial biogenesis and β-oxidation. AMPK inhibits adipogenesis via the inhibition of the early mitotic clonal expansion (MCE) phase accompanied with a reduction in early and late adipogenic factors including FAS, SREBP-1c and aP2 [162]. Therefore, the inhibition of differentiation into adipocytes by bioactive compounds from plant and herb extracts is beneficial for the loss of body fat and prevention of obesity (Table 1 and Figure 2). 

### Adipogenesis as a Possible Target against Obesity

The principal cause of obesity is energy overconsumption and/or insufficient energy expenditure; thereby, excessive food/energy intake leads to the expansion of WAT through de novo adipogenesis with the recruitment of new adipocytes (hyperplasia) and enlargement of existing adipocytes (hypertrophy). Chokeberry extract (*Aronia melanocarpa*) and its active polyphenols (seven antiadipogenic polyphenolic phytochemicals) were investigated in HFD-induced obese mice. Amygdalin and prunasin were shown to inhibit 3T3-L1 adipocyte differentiation by suppressing the expressions of PPARγ, C/EBPα, SREBP-1c, FAS, and aP2. In addition, Chokeberry extract showed in obese mice significant decreases in body weight, serum TG, and LDL-C levels and improved insulin sensitivity [163]. The effects of cinnamon (*Cinnamomum zeylanicum*) extract were examined on the inhibition of adipocyte differentiation in 3T3-L1 fibroblast cells and in male mice fed an HFD. Cinnamon extract inhibited lipid accumulation and increased adiponectin and leptin genes in 3T3-L1 cells. In in vivo experiments, cinnamon extract elevated the expression of lipolysis-related proteins (AMPK, p-ACC, and CPT-1) and decreased the expression of lipid-synthesis-related proteins (SREBP-1c and FAS) in liver tissue [164]. Corni Fructus extract (CFE), which contains Corni Fructus, Dioscoreae Rhizoma, Aurantii Fructus Immaturus, and Platycodonis Radix, was shown to suppress the differentiation of 3T3-L1 adipocytes by reducing the cellular induction of PPAR*γ*, C/EBP*α*, and lipin-1, including a significant upregulation of AMPK-*α* phosphorylation. Moreover, CFE in obese mice fed an HFD induced weight loss. Therefore, CFE has a potent antiobesity activity due to the inhibition of adipocyte differentiation and adipogenesis [165]. *Cydonia oblonga* fruit extract (COFE) was tested on adipogenesis in 3T3-L1 preadipocytes. COFE inhibited intracellular TG deposition during adipogenesis. Furthermore, COFE treatment in 3T3-L1 cells induced the upregulation of AMPK-α phosphorylation and downregulation of adipogenic transcription factors (SREBP-1c, PPARγ, and C/EBPα). COFE also reduced the mRNA expression of FAS, ACLY, aP2, and lipoprotein lipase (LPL), including increased HSL and CPT-1 in 3T3-L1 cells [51]. *Hibiscus sabdariffa* extract (HSE) was examined on adipocyte differentiation in 3T3-L1 preadipocytes. HSE significantly inhibited lipid droplet accumulation and attenuated adipogenic transcriptional factors C/EBPα and PPARγ during adipogenesis. HSE also reduced the expression of PI3K/AKT and phosphorylation and expression of MEK1/ERK during adipocyte differentiation. Taken together, HSE inhibits adipocyte differentiation through the regulation of PI3K/AKT and ERK pathways, which play pivotal roles during adipogenesis [166]. *Ilex paraguariensis* extracts (IPE) were investigated in 3T3-L1 adipocytes and HFD-fed obese Sprague Dawley (SD) rats. IPE inhibited intracellular lipid accumulation in 3T3-L1 adipocytes, increased AMPK-α, HSL, CaMKK, LKB1, PKA, C/EBPβ, IRβ, and IRS1(Tyr465), and decreased SREBP-1c, FAS, PPARγ, and IRS1 (Ser1101). Furthermore, an AMPK-α inhibitor abolished the effects exerted by IPE on intracellular lipid accumulation and HSL and FAS expression levels. In animals, IPE inhibited body weight gain and ameliorated serum cholesterol levels, and increased AMPK-α, PKA, ERK1/ERK2 (p44/p42), and UCP1 and reduced those genes of mammalian target of rapamycin, S6 kinase, SREBP-1c, ap2, FAS, Il6, adiponectin, leptin, and FABP4 in obese SD rats [167]. *Moringa oleifera* leaf petroleum ether extract (MOPEE) which has high levels of isoquercitrin, chrysin-7-glucoside, and quercitrin, was studied on lipid accumulation by in vitro and in vivo experiments. MOPEE suppressed adipogenesis in 3T3-L1 adipocytes by downregulating the expression of adipogenesis-associated proteins (PPARγ, C/EBPα and C/EBPβ, and FAS) and upregulating the expression of a lipolysis-associated protein (HSL). MOPEE also significantly increased the phosphorylation of AMPKα and ACC. In HFD-induced obese mice, MOPEE decreased body weight, epididymal, perirenal, and mesenteric fat weight, and fat tissue size, including hepatic fat accumulation. Furthermore, MOPEE also decreased TC, LDL-C, and aspartate transferase (AST). Additionally, MOPEE decreased the expression of adipogenesis-associated proteins (PPARγ and FAS) and upregulated the expression of a lipolysis-associated protein (ATGL) in the liver and epididymal fat tissue. MOPEE also increased the phosphorylation of AMPKα and ACC in the liver and epididymal fat tissue of obese mice. Therefore, MOPEE suppresses fat accumulation by inhibiting adipogenesis and promoting lipolysis [82]. A study explored the antiadipogenic effects of lyophilized *Opuntia* cladode powders (OCP) in an in vitro and an in vivo HFD-induced obesity rat model. Two OCP were tested (O. streptacantha and O. ficus-indica). OCP impaired differentiation in adipocytes (3T3 F442A) and decreased TG content and low glucose uptake, thus suggesting an antiadipogenic effect. In SD rats, OCP slightly reduced body weight gain and liver and abdominal fat weights, improved some metabolic parameters, and augmented TG excretion in the feces [168]. *Platycodon grandiflorus* extract (PGE) was investigated on pre-adipocyte 3T3-L1 differentiation, pancreatic lipase activity, and HFD-induced obese rats. PGE inhibited 3T3-L1 pre-adipocyte differentiation and fat accumulation and reduced pancreatic lipase activity. In SD rats, PGE significantly reduced plasma TC and TG levels, body weight, and subcutaneous adipose tissue weight. PGE also reduced the size of subcutaneous adipocytes [169]. *Taraxacum officinale* (Dandelion) was investigated on adipocyte differentiation and lipogenesis in 3T3-L1 preadipocytes. Leaf and root extracts and a commercial root powder (caffeic and chlorogenic acids as the main phenolic constituents) were used in the study. All extracts tested inhibited adipocyte differentiation and lipid accumulation in 3T3-L1 cells [114]. Therefore, the analyzed herbal and plant extracts play an important role during adipogenesis and lipid metabolism, supporting their therapeutic potential for the prevention and treatment of obesity (Table 1 and Figure 2).

**Table 1:** Extracts from different herbs and plants with antiobesity properties.

Name of Herbs and Plants and Method of Extraction	Type of Study	Doses and Duration	Outcomes and Side Effects (Humans)
*Allium sativum* (Garlic) Aged Garlic Extract (15–20% aqueous ethanol). Extract on the market in different brands.	Isolated human platelets stimulated with ADP	1.56 to 25% (*v*/*v*)	Inhibited platelet binding to fibrinogen by 40–70.4%, decreased PAC-1 binding to GPIIb/IIIa by 72%, and increased cAMP levels [155]. Garlic extract may cause breath and body odor, upset stomach, or heartburn.
*Andrographis paniculata* (ethanolic extract). Extract on the market in different brands.	4-week-old male C57/BL6 mice with HFD (45% kcal from fat)	2 g/kg/day, orally for a week	Attenuated cardiac hypertrophy and apoptosis, decreased ANP and BNP proteins, reduced cardiac collagen accumulation and fibrosis, inhibited COX-2, p-IκBα, and NF-κB proteins, reversed cardiac inflammation and myocardial apoptosis [152]. *Andrographis* can cause diarrhea, vomiting, rash, headache, runny nose, and fatigue.
*Aronia melanocarpa* (Chokeberry), methanol extract. Extract on the market in different brands.	3T3-L1 adipocytes and 5-week-old male C57BL/6J mice with HFD (60% kcal from fat)	In vitro: 7 polyphenols at 10 µM for 8 days. In vivo: 100 or 200 mg/kg/day, oral-ly for 8 weeks	Inhibited 3T3-L1 adipocyte differentiation, decreased body weight, serum TG, and LDL-C levels; improved insulin sensitivity [163]. Chokeberry extract can cause constipation, diarrhea, or nausea. Taking chokeberry together with drugs that slow blood clotting might increase the risk of bruising and bleeding.
*Aronia melanocarpa* (Chokeberry) Polyphenol-rich extract (aqueous extract). Extract on the market in different brands.	Human platelets stimulated with ADP	Platelet adhesion assay (range 0.5–100 µg/mL), thrombin activity (0.5–100 mg/mL), Plasmin activity (2.5, 5, 10, 20, 100 µg/mL)	Reduced ADP-activated platelet adhesion, increased overall potential of clotting and lysis, inhibited thrombin and plasmin amidolytic activity [153]. Chokeberry extract can cause constipation, diarrhea, or nausea. Taking chokeberry together with drugs that slow blood clotting might increase the risk of bruising and bleeding.
*Aronia melanocarpa* (Chokeberry), the extract was purchased from Agropharm SA (Poland).	Patients with metabolic syndrome	100 mg, three times daily for 2 months	Reduced TC, LDL-C, and TG levels, inhibited platelet aggregation (less pronounced after 2 months), decreased potential for coagulation and clot formation, beneficial changes in coagulation and fibrinolysis parameters [154]. No significant adverse effects were recorded [154]. Taking chokeberry together with drugs that slow blood clotting might increase the risk of bruising and bleeding.
*Camellia sinensis* (Green tea aqueous extract, GTAE). Extract on the market in different brands.	12-week-old male Wistar rats with HFD (50% kcal from fat)	1.1% and 2.0% GTAE for 8 weeks	Reduced body weight gain (5.6% decrease at 2.0% GTAE), prevented visceral fat accumulation (17.8% reduction at 2.0% GTAE), lowered atherogenic index (14.3% reduction at both doses), reduced protein digestion (82.6% and 84.3% at 1.1% and 2.0% GTAE, respectively) [134]. Green tea extracts can cause liver problems, and the symptoms can include yellowing of your skin or the whites of your eyes, stomach pain and nausea.
*Caralluma fimbriata* (alcohol extract). Extract on the market in different brands.	Male Wistar rats (200–220 g) with cafeteria diet	25, 50, 100 mg/kg/day for 90 days	Inhibited food intake, prevention of body weight, liver weight, and fat pad mass gains, improved serum lipid and leptin profiles, and protection against atherogenesis [136]. No adverse effects were reported [136]. *Caralluma fimbriata* can cause constipation and gas.
*Caralluma fimbriata* (40% aqueous alcohol). Extract on the market in different brands.	Male Wistar rats (170–190 g) with HFD (60 kcal% from fat)	200 mg/kg/day for 90 days	Attenuated cardiac lipids and oxidative stress, and improved antioxidant enzyme activities [27]. *Caralluma fimbriata* can cause constipation and gas.
*Caralluma fimbriata* (dry extract concentrate in gelatin capsules). Capsules on the market in different brands.	Double-blind, randomized, placebo-controlled trial	1 g/kg/day for 16 weeks	Reduced waist circumference, calorie intake, maintained body weight, reduced fat mass and BMI, and improved satiety markers [26]. Only 4 of the participants reported rash and minor gastrointestinal symptoms (bloating, loose stools) [26].
*Cinnamomum zeylanicum* (70% ethylenealcohol). Extract on the market in different brands.	3T3-L1 cells and 7-week-old male C57BL/6J mice with a normal diet with 45% fat	In vitro: 1, 3, 5, 7, 10 µg/mL for 3 days. In vivo: 1% cinnamon extract for 14 weeks	In vitro: Inhibited lipid accumulation, increased adiponectin and leptin gene expression. In vivo: Reduced lipid synthesis, increased lipolysis, decreased VLDL-C, increased HDL-C, and lowered body fat and fatty tissue accumulation [164]. No reported side effects.
*Citrullus colocynthis*, hydro-alcoholic extract (80/20, *v*/*v*). Extract on the market in different brands.	9-week-old male Sprague Dawley rats with HFD (45% kcal from fat)	50 mg/kg/day, orally for 16 weeks	Enhanced bleeding time and tPA levels, decreased PAI-1 and thromboxane B2, inhibited platelet aggregation, reversed HFD-induced increases in fibrinogen and von Willebrand factor, decreased food intake, pancreatic lipase activity, TNF-a, IL-6, and leptin, and increased adiponectin levels [34]. Colocynth can cause severe irritation of the stomach and intestine lining, bloody diarrhea, bloody urine, kidney damage, and inability to urinate. Also, can cause convulsions, paralysis, and death.
*Coffea* (Green coffee bean extract, GCBE from KPLC group: Montagne, France).	5-week-old male C57BL/6J mice with HFD (60% Kcal from fat)	Obesity induction for 4 weeks and then with extract (50, 100, 200 mg/kg/day) for 6 weeks	Reduced body weight gain, liver weight, and white adipose tissue weights. Increased adiponectin and reduced leptin. GCBE upregulated mRNA levels of PPARα, ATGL, and HSL, and downregulated adipogenesis-related genes like C/EBPα, SREBP-1c, and PPARγ. GCBE increased pAMPK expression [141]. Consuming large amounts of green coffee might cause headache, anxiety, agitation, and irregular heartbeat.
*Coffea* Arabica (aqueous extract). Not available as an extract on the market.	Male Wistar rats (160–180 g) with HFD (40% beef tallow)	Obesity induction for 8 weeks and then with extract (200 mg/kg/day) for 8 weeks	Decreased body and organ weights, reduced TC, TG, LDL-C, VLDL-C, glucose, and insulin levels, improved HOMA-IR, increased adiponectin, and reduced adipocyte hypertrophy [142]. Consuming large amounts of green coffee might cause headache, anxiety, agitation, and irregular heartbeat.
*Coffea* canephora var. robusta beans (hot-water extract). Not available as an extract on the market.	8–12-week-old male ApoE^−/−^ mice with HFD (42% kcal from fat)	At 2 weeks received 220 mg/kg/day for 14 weeks. At 4 weeks received HFD for 12 weeks	Improved fasting glucose, insulin resistance, serum leptin, urinary catecholamines, and liver triglycerides. Reduced weight gain, adiposity, and inflammatory infiltrate in adipose tissue. Recovered operational taxonomic units (alpha diversity) [157]. Consuming large amounts of green coffee might cause headache, anxiety, agitation, and irregular heartbeat.
Combination of Corni Fructus, Dioscoreae Rhizoma, Aurantii Fructus Immaturus, Platycodonis Radix (ethanol extract). Not available on the market.	3T3-L1 adipocytes and 5-week-old male C57BL/6J mice with HFD (60% kcal from fat)	In vitro: 10, 50, 100 µg/mL for 48 h. In vivo: Obesity induction for 4 weeks and then with extract (100 mg/kg/day) for 16 weeks	Inhibited the differentiation of 3T3-L1 adipocytes and expressions of PPARγ, C/EBPα, and lipin-1, increased phosphorylation of AMPK-α, and reduced weight gain in mice [165]. No side effects have been reported.
*Cydonia oblonga* (30% ethanol). Extract on the market in different brands.	3T3-L1 adipocytes	0–600 µg/mL for 8 days	Inhibited intracellular TG accumulation, induced AMPKα phosphorylation, downregulated adipogenic transcription factors (SREBP-1c, PPARγ, C/EBPα), reduced mRNA expression of FAS, ACL, aP2, LPL, and increased mRNA expression of HSL and CPT-1 [51]. No side effects have been reported.
*Cydonia oblonga* (aqueous extract). Extract on the market in different brands.	Male ICR mice (18–22 g) and male Wistar rats (300–350 g)	20, 40, 80 mg/kg/day, orally for 14 days	Prolonged bleeding and clotting times reduced pulmonary embolus mortality, increased thrombolysis, shortened ELT, reduced arterial and venous thrombus weights, decreased TXB2 and increased 6-keto-PGF1α levels [53]. No side effects have been reported.
*Ginkgo biloba* (extract obtained from Huacheng Biotech Inc. China).	2-month-old male Wistar rats with HFD (57.3% from fat)	Obesity induction for 2 months and then with extract (500 mg/kg/day), orally for 2 weeks	Reduced energy intake, epididymal adipocyte volume, and lipid accumulation. It also reduced Plin 1 and Fasn mRNA and FAS protein levels [140]. No side effects were reported [140].
*Ginkgo biloba* (unspecified extract). Extract on the market in different brands.	Male Sprague Dawley rats (200–250 g) with acute myocardial infarction	100 mg/kg/day, orally for 4 and 8 weeks	Decreased TGF-β1, MMP-2, and MMP-9 mRNA transcription levels, reduced protein levels of type I collagen, MMP-2, and MMP-9, and inhibited myocardial remodeling after AMI [156]. *Ginkgo biloba* can cause stomach upset, headache, dizziness, and allergic skin reactions. *Ginkgo* leaf extract might increase the risk of bruising and bleeding or cause arrhythmia.
*Hibiscus sabdariffa* (water extract). Extract on the market in different brands.	7–9 weeks old male C57BL/6J mice with HFD (60% kcal from fat)	1, 10, 25 mg/kg/day for 42 days	Inhibited adipogenesis via PI3-K and MAPK pathways, reduced weight gain, improved glucose tolerance and insulin sensitivity, normalized LDL-C/HDL-C ratio, reduced inflammatory state in liver, reinforced gut integrity, and prebiotic effects on gut microbiota [158]. *Hibiscus sabdariffa* can cause stomach upset, gas, and constipation.
*Hibiscus sabdariffa* (hot-water extract). Extract on the market in different brands.	3T3-L1 adipocytes	2 mg/mL for 5 days	Inhibited adipocyte differentiation through PI3K/AKT and ERK pathways, and decreased lipid droplet accumulation [166]. *Hibiscus sabdariffa* can cause stomach upset, gas, and constipation.
*Ilex paraguariensis* (Yerba mate), water extract. Extract on the market in different brands.	6-week-old male Swiss strain mice with HFD	Obesity induction for 8 weeks and then with extract (1 mg/kg) for 8 weeks	Attenuation of weight gain, decreased adiposity and epididymal fat-pad weight, restored serum levels of cholesterol, TG, LDL-C, and glucose [75]. Yerba mate can cause insomnia, upset stomach, increased heart rate, and others.
*Ilex paraguariensis* (Yerba mate), water extract. Available on the market in different brands.	6-week-old male C57BL/6J mice with HFD (60% kcal from fat)	Obesity induction for 6 weeks and then with extract (0.5, 1, or 2 g/kg/day) for 4 weeks	Reduced body weight gain, lower adipose tissue, decreased serum cholesterol, TG, and glucose levels [76]. Yerba mate can cause insomnia, upset stomach, increased heart rate, and others.
*Ilex paraguariensis* (Yerba mate), 15% etanol extract. Available on the market in different brands.	6-week-old male Sprague Dawley rats with HFD (40% kcal from fat)	Daily supplementation of extract, 0.24% (*w*/*w*) for 60 days	Reduced body weight, visceral fat, blood and Hepatic lipid levels, improved glucose and insulin levels, enhanced AMPK phosphorylation, increased UCP2 and UCP3 expression [144]. Yerba mate can cause insomnia, upset stomach, increased heart rate, and others.
*Ilex paraguariensis* (Yerba mate), water extract. Available on the market in different brands.	Early weaned Wistar rats	1 g/kg BW/day, gavage for 30 days	Reduced adipose mass (retroperitoneal and epididymal), total body fat, subcutaneous fat, visceral adipocyte area, TG, and hypothalamic NPY content; restored central leptin resistance, hyperphagia, and higher hypothalamic SOCS-3 content [145]. Yerba mate can cause insomnia, upset stomach, increased heart rate, and others.
*Ilex paraguariensis* (Yerba mate), water extract (capsules). Available on the market in different brands.	A randomized, double-blind, placebo-controlled clinical trial on obese Korean adults	3 g/day for 12 weeks	Decreased body fat mass, percent body fat, and WHR [147]. Yerba Mate supplementation did not cause any adverse side effects [147].
*Ilex paraguariensis* (Yerba mate), water extract. Available on the market in different brands.	8-week-old male Wistar rats with HFD (45% kcal from lard fat)	100 mg/day in 3rd month of age and 200 mg/day in 4th month of age, daily for 2 months	Reduced hypothalamic IKK phosphorylation and NF-κB p65 expression, increased IκBα and AdipoR1 expression, reduced IL-6 levels, increased IL-10/TNF-α ratio, and reduced low-grade inflammation [151]. Yerba mate can cause insomnia, upset stomach, increased heart rate, and others.
*Ilex paraguariensis* (Yerba mate), water extract. Available on the market in different brands.	3T3-L1 adipocytes and 8-week-old male Sprague Dawley rats with HFD (507.6 kcal/100 g)	In vitro: 10, 50, 100 µg/mL for 7 days. In vivo: 500 mg/kg/day for 8 weeks	In vitro: Suppressed lipid accumulation, Increased AMPK, HSL, CaMKK, LKB1, PKA, C/EBPβ, Irβ, IRS1 (Tyr465), decreased SREBP-1c, FAS, PPARγ, and IRS1 (Ser1101). In vivo: suppressed body weight gain, improved serum cholesterol levels, increased AMPK, PKA, ERK1/ERK2, UCP1, reduced mTOR, S6K, SREBP-1c, ap2, FAS, IL-6, adiponectin, leptin, and FABP4 [167]. Yerba mate can cause insomnia, upset stomach, increased heart rate, and others.
*Moringa oleifera * (70% ethanol extract). Extract on the market in different brands.	Male albino rats (100 ± 20 g) with HFD (58% fat) and overweight/obese female patients	In vivo: Obesity induction for 2 months and then with extract (200 and 400 mg/kg/day) for 1 month; patients: gelatine capsules (400 mg/day) for 8 weeks	In rats, reduced final weight, adiposity index, glucose, insulin, and HOMA-IR. Increased R-QUICKI, adiponectin, omentin, GLUT4, and PPARα expression. Reduced leptin and vaspin. Suppressed FAS and HMG-CoA reductase. In patients, reduced BMI, TC, and LDL-C [81]. *Moringa oleifera* is likely safe when the leaves, fruit, and seeds are eaten as food.
*Moringa oleifera* leaf petroleum ether extract (MOPEE). Extract on the market in different brands.	3T3-L1 adipocytes and 7-week-old male C57BL/6J mice with HFD (60% kcal from fat)	In vitro: 0, 50, 100, 200, and 400 µg/mL for 24 h. In vivo: 0.125, 0.25, 0.5 g/kg/day for 14 weeks	In vitro: Inhibited adipogenesis in a dose-dependent manner. Downregulated PPARγ, C/EBPα, C/EBPβ, FAS. Upregulated HSL, AMPKα, and ACC phosphorylation. In vivo: Decreased body weight, fat pad weight, and hepatic fat accumulation. Reduced TC, LDL-C, and AST levels. Downregulated PPARγ and FAS. Upregulated ATGL, AMPKα, and ACC phosphorylation [82]. *Moringa oleifera* is likely safe when the leaves, fruit, and seeds are eaten as food.
*Moringa oleifera* (methanol extract from leaves). Extract on the market in different brands.	3-month-old male Wistar rats with HFD	200 and 400 mg/kg/day for 12 weeks	Alleviated serum biochemical abnormalities, balanced antioxidant status, and reestablished normal heart histology [150]. *Moringa oleifera* is likely safe when the leaves, fruit, and seeds are eaten as food.
*Opuntia streptacantha* and *Opuntia* *ficus-indica*. *Opuntia* young cladode powders. Not available on the market.	3T3-F442A adipocytes and 6-week-old male Sprague Dawley rats with HFD (60% kcal from fat)	In vitro: 1, 10, 100 μg/mL for 10 days. In vivo: 0.5% *w*/*w* for 8 weeks	In vitro: Impaired adipocyte differentiation and decreased TG, and reduced glucose uptake. In vivo: Slightly reduced body weight gain, liver and abdominal fat weights. Increased TG excretion in feces [168]. *Opuntia ficus-indica* can cause nausea, bloating, mild diarrhea, increased quantity and frequency of stools, and headache.
*Platycodon* *grandiflorus* (ethanol extract). Extract on the market in different brands.	Randomized, double-blind, placebo-controlled clinical trial on overweight or moderately obese adults	571 mg, 1142 mg, 2855 mg (in tablets) per day for 12 weeks	Decreased body fat mass and body fat percentage, reduced total abdominal and subcutaneous fat areas, increased muscle mass [148]. Side effects not reported.
*Platycodon grandiflorus* (water extract). Extract on the market in different brands.	3T3-L1 preadipocytes and 8-week-old male Sprague Dawley rats with HFD (59.8% kcal from fat)	In vitro: various concentrations (10–50 mg/mL). In vivo: 150 mg/kg/day for 7 weeks	Inhibited 3T3-L1 preadipocyte differentiation and fat accumulation. Decreased pancreatic lipase activity. In vivo: Reduced plasma TC) and TG levels, decreased body weight and subcutaneous adipose tissue weight, reduced size of subcutaneous adipocytes, repressed up-regulation of FABP mRNA in subcutaneous adipose tissue [169]. Side effects not reported.
*Punica granatum* (Pomegranate), ethanol:water 70:30. Extract on the market in different brands.	6-week-old male C57BL/6 mice with HFD (45% of total fat)	0.1 g/kg/3 days per week 0.2 for 12–14 weeks	Increased energy expenditure, reduced chronic inflammation and insulin resistance, promoted browning and thermogenesis in adipose tissue, reduced inflammatory markers, increased the reductive potential [104]. Some people have experienced sensitivity to pomegranate extract such as itching, swelling, runny nose, and difficulty breathing.
*Salvia miltiorrhiza* (75% etanol extract). Extract on the market in different brands.	8–9-week-old male Sprague Dawley rats with HFD (45% kcal from fat)	0.675, 1.35, 2.70 g/kg/day for 8 weeks	Reduced body weight, body fat index, serum lipid level, hepatic lipid accumulation, and adipocyte vacuolation. Improved gut integrity and lipid metabolism altered gut microbiota composition [110]. *Salvia miltiorrhiza* can cause upset stomach, itching, and reduced appetite.
*Taraxacum officinale* (95% ethanol extract). Extract on the market in different brands.	Porcine pancreatic lipase and 7-week-old male ICR mice	In vitro: 50–250 µg/mL. In vivo: 400 mg/kg single dose for 240 min	In vitro: inhibited pancreatic lipase activity. In vivo: decreased plasma TG levels and reduced AUC of plasma TG response curve [113]. *Taraxacum officinale* can cause allergic reactions, stomach discomfort, diarrhea, or heartburn in some people.
*Taraxacum officinale* (leaf and root extracts in ethanol 60%). Extract on the market in different brands.	3T3-L1 adipocytes	300–600 μg/μL for 6 days	Inhibited adipocyte differentiation, reduced lipid and TG accumulation, regulated expression of genes and long non-coding RNAs involved in adipogenesis and lipid metabolism [114]. *Taraxacum officinale* can cause allergic reactions, stomach discomfort, diarrhea, or heartburn in some people.
*Theobroma cacao* (aqueous extract). Extract on the market in different brands.	Wistar rats (250 ± 20 g) with HFD (45% kcal) and 20% fructose	Obesity induction for 5 weeks and then with 100%, 10%, 1% pellet for 5 weeks	Decreased body weight by 39%, systolic blood pressure by 27%, triglycerides by 55%, TC by 24%, LDL-C by 37%, and TG/HDL-C ratio by 54% [42]. Cocoa can cause allergic skin reactions, migraine headaches, nausea, stomach discomfort, constipation, and gas. Eating large amounts can cause caffeine-related side effects such as nervousness, increased urination, sleeplessness, and a fast heartbeat.

## 5. Plant and Herb Extracts Targeting Dyslipidemia and Adipokines in Obesity

Obesity-related dyslipidemia is considered as an atherogenic lipoprotein phenotype and one of the major risk factors for ischemic heart disease. The main manifestations of dyslipidemia include elevated plasma concentrations of TC, LDL-C and TGs, and low levels of HDL-C, which are important factors in hypertension and CVD [170]. Visceral obesity promotes insulin resistance in part mediated by high levels of FFAs and adipokines dysregulation. Adipokines such as leptin, resistin, and retinol-binding protein 4 increase insulin resistance, whereas adiponectin with anti-inflammatory and antilipogenic effects increases insulin sensitivity. In addition, in obesity, pro-inflammatory mediators (leptin, resistin, IL-6, and TNF-α) may promote adipose tissue dysregulation and systemic insulin resistance [1].

In male SD rats fed with HFD were investigated the effects of a high hydrostatic pressure extract of garlic (HEG) on HDL-C level and hepatic apolipoprotein A-I (apoA-I) gene expression. In animals treated with HEG, plasma TC, TG and LDL-C levels were significantly decreased, while the plasma HDL-C level and mRNA level of hepatic apoA-I were significantly increased. Furthermore, HEG upregulated the gene expression of ATP-binding cassette transporter A1 (ABCA1) and lecithin-cholesterol acyl transferase (LCAT) in obese rats [171]. LVH is a risk factor for cardiovascular morbidity and mortality [172]. The effects of three *Camellia sinensis* teas (green, red, and white) were studied on LVH and insulin resistance in LDLr^−/−^ mice fed an HFD. The teas partially prevented hyperlipidemia, increased HDL-C, reduced insulin resistance and CRP levels, and completely prevented LVH in LDLr^−/−^ mice fed an HFD [173]. A systematic review and meta-analysis reported that green tea extract (GTE) significantly reduced TC, LDL-C, fasting blood sugar, hemoglobin A1c (HbA1c), and DBP, while increasing HDL-C [174]. Corni Fructus extract (CFE) was administered in a rat model of diet-induced hypercholesterolemia, and the extract inhibited the elevation of both systolic and diastolic (BP), and lowered serum TC levels with a decrease in esterified cholesterol. Additionally, the protein expressions of SREBP-2 and PPARγ were elevated, indicating that CFE would activate FA oxidation [47]. *Cydonia oblonga* extract (COE) with flavonoids (>60%) from leaves and fruit was analyzed on the blood lipid and antioxidant effects using hyperlipidaemic rat models. The flavonoids from COE significantly reduced serum TC, TG, LDL-C, ALT and AST and increased HDL-C. Flavonoids improved the activity of SOD and GSH-Px in hepatic tissues and reduced malondialdehyde acid (MDA) [52]. In rats fed with HFD, *Ilex paraguariensis* (yerba mate) extract reduced serum TG and TC and decreased the atherogenic index [175]. *Ilex paraguariensis* extract from leaves was investigated on hyperlipidemia induced in hamsters by an HFD. Yerba mate extract significantly reduced body-weight gain and lowered serum lipid levels; meanwhile, Yerba mate treatment increased antioxidant enzyme activity, ameliorated LPL and hepatic lipase activities in serum and liver, upregulated mRNA expression of PPARα, and downregulated mRNA expression of SREBP-1c and ACC in the liver. Therefore, Yerba mate extract regulates the expression of genes involved in lipid oxidation and lipogenesis [176]. Moreover, Yerba mate infusions were also studied in dyslipidemic individuals over 18 years of age (men and women). Yerba mate tea showed a significant increase in ferric reducing antioxidant potential and decreased glutathione concentrations but no significant changes in lipid hydroperoxide (LOOH), protein carbonyl, and paraoxonase-1 levels; thereby, Yerba mate tea increases plasma and blood antioxidant protection in patients with dyslipidemia [177]. However, a systematic review and meta-analysis found no differences in TC, LDL-C, HDL-C, and TG levels when comparing the yerba mate and control groups. The authors concluded that because the results are based on small inconclusive studies, more research is needed to confirm these findings [178]. *Moringa oleifera* leaves are used in India as a hypocholesterolemic agent in obese patients. A study reported that administration of the crude leaf extract of *Moringa oleifera* along with HFD reduced the HFD-induced increases in serum, liver, and kidney cholesterol levels. Furthermore, the crude extract increased serum albumin [83]. *Nigella sativa* has been used for the treatment and prevention of hyperlipidemia. A study analyzed different preparations reported of *Nigella sativa* including seed powder (100 mg-20 g daily), seed oil (20–800 mg daily), thymoquinone (3.5–20 mg daily), and seed extract (methanolic extract especially), and found that these preparations of *Nigella sativa* reduce plasma concentrations of TC, LDL-C, and TG, but the effect on HDL-C was not significant. The authors concluded that lipid-modifying properties of *Nigella sativa* could be attributed to the suppression of intestinal cholesterol absorption, reduced hepatic cholesterol synthesis, and up-regulation of LDL-C receptors [85]. Cholesterol reduction is critical for the prevention of CVD. *Opuntia ficus-indica* extract (OFIE) was tested on the inhibitory activity of pancreatic lipase enzyme (in vitro) and on hypercholesterolemia induced in mice by intraperitoneal administration of Triton WR-1339 (in vivo). The extracts significantly decreased blood cholesterol levels and inhibited pancreatic lipase activity. Therefore, OFIE prevents hypercholesterolemia by pancreatic lipase inhibition, partly attributed to its polyphenolic compounds [90]. *Platycodon grandiflorus* extract (PGE) was investigated in obese mice. The extract reduced body weight gain and improved plasma lipid profiles. Furthermore, leptin was significantly reduced whereas adiponectin was elevated. PGE also downregulated lipogenic gene (LPL, ACC, and FAS) expression and increased lipolysis genes (CPT-1, HSL, and UCP2) in WAT and liver. Moreover, PGE inhibited adipogenic transcriptional factors, such as PPARγ, C/EBPα, and SREBP-1c [97]. Another study related to *Platycodon grandiflorus* root extract in HFD-induced obese mice reported that the extract exhibited antioxidant activity; meanwhile, in calf pulmonary arterial endothelial cells, both oxLDL-C-induced cell death and lactate dehydrogenase release were inhibited. In obese mice treated with *Platycodon grandiflorus* root extract, antioxidant proteins were increased, and plasma and hepatic lipid levels were reduced, thus demonstrating its beneficial effects on hyperlipidemia [98]. The representative adipokines secreted from adipose tissues with an increased plasma leptin, resistin, and TNF-α, and a reduced plasma adiponectin are related to systemic insulin resistance [1]. PGE in male ICR mice fed an HFD markedly attenuated food intake, epididymal fat weight, body weight, adipocyte size, and blood glucose levels while maintaining serum levels of adiponectin, resistin, leptin, fructosamine, and TGs. PGE also up-regulates adiponectin and down-regulates TNF-α, and leptin in fat tissue. In L6 muscle cells, the extract elevated insulin-stimulated glucose uptake [99]. Taken together, the evidence from experimental and clinical studies suggests that plant extracts have lipid-lowering effects and adipokines regulation, which may be suitable for the prevention and treatment of obesity (Table 2 and Figure 2).

## 6. Plant and Herb Extracts against to Insulin Resistance, Hyperglycemia, and Diabetes

Several factors contribute to the growth of obesity and T2D, such as unhealthy eating habits, physical inactivity, sedentary lifestyle, increased stress, and environmental factors, which are typical of global urbanization and modern society in the world [1]. Increased BMI and excessive visceral adipose tissue are known to be associated with insulin resistance and beta cell dysfunction, which can lead to glucose intolerance and T2D. Obesity plays an important role in the elevated prevalence of T2D, which is characterized by insulin resistance in several tissues, including muscle, liver, and adipose tissue. T2D is manifested by low insulin secretions from β-cells and peripheral insulin resistance, including high levels of fatty acids, which promote systemic inflammation. Obesity can cause a chronic low-grade inflammatory state with high levels of cytokines such as TNF-α, IL-1β, monocyte chemoattractant protein-1 (MCP-1), and IL-6, which in part contributes to the pathogenesis of insulin resistance, promoting a reduction in glucose uptake in insulin-dependent tissues, which increases blood glucose levels, β-cell dysfunction in pancreas and results in endocrine dysfunction of adipose tissue. One of the signaling pathways involved in the inflammatory mechanisms is the activation of JNK1 by TNF-α, which results in serine phosphorylation of insulin receptor substrate 1 (IRS1) and impairs insulin signaling and subsequent insulin resistance [1,179].

In addition to obesity and T2D hyperglycemia is a known risk factor for the development of several health disorders such as oxidative stress and cardiovascular diseases. Antidiabetic effects of pregnane glycosides, alkaloids, and tannins/gallic tannins derived from *Caralluma fimbriata* have been reported [25]. The effects of *Caralluma fimbriata* extract (CFE) on insulin resistance and oxidative stress in HFD-fed Wistar male rats were investigated. The administration of CFE in obese animals resulted in a significant improvement in plasma glucose, insulin, leptin, and TGs. CFE also prevented high levels of lipid peroxidation and protein oxidation, low GSH levels, and low activities of enzymatic antioxidants [180]. Cinnamon extracts have been used to treat blood glucose. *Ceylon cinnamon* extract (CCE) was tested on carbohydrate digestion and post-meal blood glucose reduction in vitro (enzymatic assays) and in vivo (starch tolerance tests in rats), including 18 healthy female and male volunteers. In an in vitro study, CCE inhibited pancreatic α-amylase activity and reduced the glycemic response to starch in a dose-dependent manner in rats. In healthy volunteers, CCE lowered the area under the curve of glycemia with no changes in insulin secretion [30]. *Citrullus colocynthis* has been used against diabetes. Colocynth fruit extract was investigated on insulin action using 3T3-L1 adipocytes. Extracts of seed and pulp enhanced insulin-induced GLUT4 translocation and increased insulin-stimulated cellular glucose uptake. Moreover, extracts of pulp enhanced insulin-induced PKB phosphorylation without affecting phosphorylation of the insulin receptor [181]. With respect to this, compounds from *Citrullus colocynthis*, such as the 2-O-β-D-glucopyranosyl forms of cucurbitacins J/K, I, E, and B, and cucurbitacins I, B, and E, are speculated to be responsible for the insulin-enhancing activity [173]. However, a systematic review and meta-analysis in diabetic patients reported that *Citrullus colocynthis* does not have a significant effect on fasting blood sugar, HbA1c, LDL-C, TC, and TG indices, but increases HDL-C. These findings could be related to the relatively low quality of articles and the small number of included studies [33]. Corni Fructus extract (CFE) was analyzed on blood glucose and insulin resistance in db/db mice. CFE suppressed an increase in blood glucose levels during the oral glucose tolerance test. In addition, CFE lowered final fasting serum glucose and TG in diabetic mice. The mRNA expression of adiponectin, GLUT4, and PPARγ in adipose tissue was higher in diabetic mice treated with CFE [48]. In another study, Corni Fructus extract was evaluated on kidneys of diabetic db/db mice. The activities of XO and SOD were significantly higher in diabetic mice with CFE; meanwhile, the activities of CAT and GST were lowered in the diabetic mice with CFE. Additionally, the mRNA expression of eNOS in kidneys was reduced in the diabetic mice with the extract. Therefore, Corni Fructus extract has antioxidative actions that contribute to its renoprotective effects on diabetic nephropathy [182]. *Ginkgo biloba* extract (GBE) leaves (water and 12% ethanol extracts) from four different trees (1 and 2—males and 3 and 4—females) were analyzed on phenolic profile, antioxidant activity, and the potential in vitro inhibitory properties on α-amylase, α-glucosidase, and ACE enzymes, which are related to diabetes and hypertension. Aqueous extracts had higher phenolic contents than ethanolic extracts. ACE activity was only inhibited by ethanolic extracts. The results showed a strong correlation between total phenolics and α-glucosidase inhibitory activity, and to a lesser degree, a positive correlation between total phenolics and α-amylase inhibitory activity [57]. Egb761, a *Ginkgo biloba* extract, has antioxidant and antiplatelet aggregation effects. The effect of Egb761 and its major subcomponents (bilobalide, kaempferol, and quercetin) on preventing atherosclerosis was analyzed in vitro using a rat model of T2D. Egb761 dose-dependently reduced the intima–media ratio and proliferation of VSMCs and promoted greater apoptosis in obese rats with T2D. In an in vitro model, Egb761 also decreased the proliferation and migration of VSMCs. Glucose and circulating adiponectin levels were ameliorated, and plasma hs-CRP concentrations were reduced in obesogenic and diabetic rats. Moreover, caspase-3 activity and DNA fragmentation were increased while monocyte adhesion and ICAM-1/VCAM-1 levels were decreased with Egb761 in obese rats with T2D. The bioactive compounds of Egb761, kaempferol, and quercetin decreased VSMC migration and elevated caspase activity [58]. Green coffee extract (GCE) was evaluated for 10 weeks on glycemic indices, inflammation, and oxidative stress in individuals with T2D and overweight/obesity. GCE supplementation reduced body weight and BMI. In addition, patients with GCE had lower fasting blood glucose (FBG) concentration, but there was no effect on insulin levels and HOMA-IR. However, there were significant improvements in SBP, TG level, HDL-C, and TG-to-HDL-C ratio. GCE supplementation did not affect DBP, LDL-C, or TC, including MDA levels. GCE significantly decreased the hs-CRP levels in patients with T2D and overweight/obesity. Therefore, GCE possesses beneficial effects on lipid profile and inflammation in individuals with T2D and overweight/obesity [183]. *Hibiscus sabdariffa* polyphenolic extract (HPE) was analyzed on the T2D rat model. HPE decreased hyperglycemia and hyperinsulinemia, including serum TG, cholesterol, and the ratio of LDL-C/HDL-C. Moreover, HPE significantly reduced the plasma advanced glycation end product (AGE) formation and lipid peroxidation in T2D rats. Furthermore, HPE inhibits the expression of connective tissue growth factor (CTGF) and receptor of AGE (RAGE). HPE retrieved the weight loss found in T2D rats [184]. Extracts of two varieties (red and white) of *Hibiscus sabdariffa* (Roselle) calyces were evaluated on carbohydrate hydrolyzing enzymes (α-amylase and α-glucosidase). The extracts inhibited the α-amylase and α-glucosidase activities in vitro, but the red variety exhibited higher α-glucosidase inhibitory activity than the white variety, while the white variety showed higher α-amylase inhibitory activity than the red variety. Additionally, the red variety possesses higher antioxidant capacity, which appears to be more potent compared to the white variety [70]. *Hibiscus sabdariffa* extract (HSE) was evaluated on the mechanism of adipogenesis and complications of obesity-related insulin resistance in HFD-induced obese SD rats. HSE reduced food intake, body weight, lipid profiles, lipid peroxidation, inflammatory cytokines, serum leptin, insulin and duodenal glucose absorption, while it significantly elevated the glucose uptake of adipose tissue and muscle. Moreover, HSE prevents lipid accumulation by inhibiting the differentiation of 3T3-L1 adipocytes through the downregulation of genes involved in adipogenesis [69]. Roasted mate tea consumption (*Ilex paraguariensis*) was evaluated on the glycemic and lipid profiles of patients with T2D or pre-diabetes. Mate tea consumption reduced the levels of fasting glucose, HbA1c, and LDL-C of T2D patients. However, the consumption of mate tea did not change the intake of total energy, carbohydrate, protein, cholesterol, and fiber. In addition, mate tea consumption together with nutritional counseling reduced significantly the levels of LDL-C, HDL-C, and TG. Therefore, mate tea consumption ameliorated the glucose levels and lipid profile of T2D patients [77]. *Moringa oleifera* has beneficial properties to reduce the risk of chronic metabolic diseases such as T2D. One study investigated capsules of *Moringa oleifera* dry leaf powder in subjects with prediabetes for 12 weeks. *Moringa oleifera* improved FBG and HbA1c. There were no significant changes in microbiota, hepatic and renal function markers, or appetite-controlling hormones, such as glucagon-like peptide 1 (GLP-1), ghrelin, and peptide YY (PYY) [84]. *Opuntia ficus-indica* var. saboten (OFS) dried powder extract was investigated using in vitro and in vivo models. OSF inhibited α-glucosidase activity in vitro and intestinal glucose absorption in db/db mice. In L6 muscle cells, OFS elevated dose-dependent glucose uptake, stimulated AMPK and p38 MAPK phosphorylations, and increased GLUT4 translocation to the cell membrane. OFS treatment in db/db mice dose-dependently prevented hyperinsulinemia, hyperglycemia, and glucose tolerance, including insulin resistance and quantitative insulin sensitivity check index. OFS ameliorated pancreatic function through elevated β-cell mass in db/db mice [185]. Another study of *Opuntia ficus-indica* extract (OFIE) prepared from the cladodes and a proprietary stem/fruit skin-blend was tested on blood glucose and plasma insulin in normal rats. OFIE significantly lowered blood glucose levels and significantly elevated basal plasma insulin levels, suggesting a direct action on pancreatic β-cells [92]. The flowering part of *Punica granatum* has been recommended in Unani literature for the treatment of diabetes. *Punica granatum* flower extract (PGFE) was analyzed on hyperglycemia in vivo and in vitro. PGF extract markedly reduced plasma glucose levels (postprandial hyperglycemia) in non-fasted Zucker diabetic fatty rats (a genetic model of obesity and T2D). In vitro, PGFE had a potent inhibitory effect on α-glucosidase activity [105]. *Salvia miltiorrhiza* extract (SME) was investigated on the expression of VEGF induced by high concentration of glucose in HMEC-1 cells, in which mitochondrial uncoupling protein 2 (UCP2) was knocked down by using UCP2 siRNA. HMEC-1 cells with 30 mM glucose resulted in a significant increase in the expression of VEGF mRNA, and high levels of ROS. SME significantly decreased VEGF mRNA and ROS formation in HMEC-1 cells with 30 mM glucose. Interestingly, the knockdown of UCP-2 abolished the reduction in VEGF expression and ROS formation by SME. Therefore, SME has antioxidant effects and can be used for the treatment of diabetic chronic vascular complications [109]. The antidiabetic potential of fresh and shade-dried leaves of *Taraxacum officinale* was investigated. The extract of shade-dried *Taraxacum officinale* leaves demonstrated potent antidiabetic activity in a dose-dependent manner by targeting α-amylase and α-glucosidase, having great potential to suppress post-prandial glucose rise and for better management of diabetes [116]. The plant extracts described in this section have antidiabetic effects because they improve glycemic control and lipid profile, postprandial hyperglycemia, insulin resistance, inflammatory cytokines, and oxidative stress in diabetic models. Therefore, their consumption combined with nutritional intervention could be a good strategy to decrease plasma glucose levels and lipid parameters in individuals with pre-diabetes and diabetes, which may reduce their risk of developing metabolic disorders and coronary artery disease (Table 3 and Figure 2).

## 7. Plant and Herb Extracts with Antihypertensive Effects

Obesity-associated hypertension is well documented in children and adults and in both sexes. Excess weight gain (especially increased visceral adiposity) is a major cause of hypertension and accounts for 65% to 75% of the risk for human primary hypertension and causes a cascade of associated cardiorenal and metabolic disorders. The mechanisms involved in obesity-associated hypertension are complex and include [1] physical compression of the kidneys from excess fat in and around the kidneys, [2] sympathetic nervous system (SNS) overactivation, [3] activation of the renin–angiotensin–aldosterone system (RAAS), [4] dysregulation in adipose tissue-secreted cytokines, such as leptin, insulin, resistance, TNF-α, and IL-6, [5] systemic insulin resistance, [6] endothelial dysfunction, and [7] structural and functional renal changes. In addition, SNS overactivation promotes elevations in heart rate, cardiac output, and renal tubular sodium reabsorption, which occur due to α-adrenergic and β-adrenergic receptor stimulation and indirectly through activation of other systems (e.g., RAAS) [186,187]. Weight loss is the main goal of reducing obesity-related hypertension, and current therapeutic approaches address the metabolic consequences of obesity, including dyslipidemia, inflammation, and diabetes.

The consumption of aged black garlic (ABG) is associated with improvements in several CVD risk factors. ABG extract along with dietary recommendations was analyzed on CVD risk factors in subjects with moderate hypercholesterolemia. The use of ABG extract for 6 weeks reduced DBP, particularly in men with a DBP > 75 mm Hg [188]. *Andrographis paniculata* extract (APE) was investigated using chronic intraperitoneal infusions by osmotic pumps in spontaneously hypertensive rats (SHRs) and Wistar–Kyoto (WKY) rats. APE significantly reduced the SBP of both SHRs and WKY rats. Plasma ACE activity and thiobarbituric acid (TBA) were significantly reduced in SHRs treated with APE [189]. In a meta-analysis of controlled clinical trials, berry extract from *Aronia melanocarpa* (chokeberry) was tested for an average of 6–8 weeks on TC and BP. Daily supplementation with berry extract significantly reduced SBP and TC, mainly in adults over the age of 50 years [190]. According to epidemiological studies, green tea (*Camellia sinensis*) consumption has protective effects against CVD. Green tea extract (GTE) with Ang II (induces endothelial dysfunction) were investigated for 13 days on arterial hypertension with high oxidative stress in male SD rats. GTE blunted the increased BP, LV mass index, media-to-lumen ratio, and hydroperoxide radicals, including HO-1, p22phox, and SOD-1 mRNA in the aorta caused by Ang II [191]. In a systematic review and meta-analysis of randomized clinical trials, GTE was analyzed in 20 human randomized clinical trials comprising 1536 participants. GTE significantly reduced SBP, TC, and LDL-C. Adverse events reported were elevated BP, rash, and abdominal discomfort [192]. In a crossover randomized clinical trial, green tea was analyzed on BP, endothelial function, inflammatory activity, and metabolic profile in obese prehypertensive women. Participants received three capsules daily containing 500 mg of green tea extract (GTE) for 4 weeks, with a washout period of 2 weeks between treatments. Each GTE capsule had 260 mg of polyphenols. After 4 weeks of GTE supplementation, there was a significant reduction in SBP at 24 h, daytime, and nighttime [193]. The intake of cocoa extract, which consisted of 1.4 g (415 mg flavanols) before and after 4 weeks of daily intake, was evaluated on postprandial cardiometabolic effects. The consumption of cocoa extract within an energy-restricted diet for 4 weeks showed a greater reduction in postprandial AUC of SBP compared to the control group and independently of body weight loss [41]. Leaf extract of *Ginkgo biloba* (Egb761) was investigated on hypertension with hypercholesterolemia-induced renal injury in rats. Hypertension was caused by L-N(G)-nitroarginine methyl ester (L-NAME), and hypercholesterolemia was induced by a diet with 1% cholesterol. Egb761 exhibited a progressive reduction in the SBP, and mean arterial BP. Moreover, Egb761 decreased the excess of MDA and nitrite levels and recovered the low levels of intracellular reduced glutathione (GSH) caused by hypertension with hypercholesterolemia in the renal tissue. Furthermore, hypertension with hypercholesterolemia increased the expression of TNF-α, IL-6, and IL-1β levels in renal tissues and was inhibited by treatment with Egb761. Chronic hypertension with hypercholesterolemia induced the inhibition of endothelial nitric oxide synthase (eNOS) and activation of inducible NO synthase (iNOS), but Egb761 activated eNOS and inhibited iNOS in the kidney tissues. Therefore, these findings suggest that Egb761 protects against hypertension with hypercholesterolemia-induced renal injury [59]. A new component group of *Ginkgo biloba* leaves (GBLCG), mainly composed of quercetin, kaempferol, and isorhamnetin, was investigated on reducing BP and ameliorating myocardial hypertrophy in SHRs. Total terpenoid lactones of GBLCG might be a novel cocrystal composed of Ginkgolide (A, B, C, J) and bilobalide. GBLCG had hypotensive activity and improved myocardial hypertrophy. These effects could be due to the promoting of NO synthesis and release in endothelial cells, reducing oxidative stress, and inhibiting platelet aggregation [194]. Green coffee bean extract (GCE) has protective effects against hypertension in both SHRs and humans. A study investigated the dose–response relationship of GCE in 117 male subjects with mild hypertension. After 28 days of using GCE, the decrease in SBP and DBP was statistically significant compared with the placebo group. Therefore, GCE has antihypertensive effects in patients with mild hypertension [64]. It has been reported that *Hibiscus sabdariffa* can reduce BP in human and animal studies. The extract of the dried calyx of *Hibiscus sabdariffa* (HS) and Hibiscus anthocyanins (Has) were investigated on left-ventricular myocardial capillary length and surface area in SHRs. HS consumption significantly decreased SBP, DBP, and LV mass in a dose-dependent fashion, but it did not affect the heart rate. HS also significantly increased the surface area and length density of myocardial capillaries and length density. Myocyte nuclear volume was reduced in rats with HS. There was an insignificant decrease in SBP and DBP with HA ingestion. This study showed that HS ingestion improves myocardial capillarization in SHRs through structural alterations linked to a reduction of myocardial mass and the promotion of new vessel formation [195]. *Hibiscus sabdariffa* extract (HSE) was investigated on RAAS in mild to moderate essential hypertensive Nigerian subjects. After 4 weeks of treatment with HSE (150 mg/kg/day), the extract significantly (*p* < 0.001) reduced plasma aldosterone; meanwhile, serum ACE and plasma renin activity did not change significantly. The effects observed could be related to the presence of anthocyanins in the extract [196]. *Hibiscus sabdariffa* calyces (HSC) extract was analyzed on BP, vascular function, and other cardiometabolic risk factors in men with 1% to 10% CVD risk. The consumption of aqueous extract of HSC significantly increased in % flow-mediated dilatation of the branchial artery, and there was no significant decrease in SBP and DBP, a non-significant increase in urinary and plasma nitric oxide (Nox) and reduced levels of plasma insulin and serum glucose, including TG and CRP. There was a significant improvement in the area under the systemic antioxidant response curve, and consumption of the HSC extract showed no significant changes in arterial stiffness [197]. HS extract was studied on isolated mesenteric arteries from normotensive (Wistar and WKY) and SHRs. HS extract caused a concentration-dependent relaxant effect on mesenteric artery rings of SHRs (EC50 = 0.83 ± 0.08 mg/mL), WKY (EC50 = 0.46 ± 0.04 mg/mL) and Wistar rats (EC50 = 0.44 ± 0.08 mg/mL) pre-contracted with phenylephrine (10 µM). HS extract of 2 mg/mL significantly reduced the peak of the L-type calcium current seen in cardiac myocytes by 24%. HS extract did not promote a membrane hyperpolarization of smooth muscle cells, which could suggest an absence of a direct effect on background potassium current. The authors concluded that HS extract probably implicates a vasorelaxant effect on small resistance arteries, which does not depend on the endothelium, and the reduction in L-type calcium current is part of this effect [71]. *Nigella sativa* seed extract (100 and 200 mg twice a day) supplement was evaluated in patients with mild hypertension. After 8 weeks, SBP and DBP values were statistically significantly reduced in a dose-dependent manner. The extract also caused a significant decline in the level of TC and LDL-C [87]. *Platycodon grandiflorus* (PG) is used to reduce inflammation and lower BP in the Chinese population. *Platycodon grandiflorus* root was tested for inhibiting Ang II-induced IGF-IIR activation and apoptosis pathway in H9c2 cells and SHRs. The crude extract of PG significantly inhibited the Ang II-induced IGFIIR signaling to avoid H9c2 cells apoptosis. PG extract suppressed Ang II-dependent JNK activation and SIRT1 degradation to decrease IGF-IIR activity. Additionally, PG maintained SIRT1 stability to improve HSF1-mediated IGF-IIR suppression, which prevents H9c2 cells apoptosis. In SHRs, PG markedly decreased this apoptotic pathway in the heart tissues; thus, PG could be considered for the treatment of heart diseases in hypertensive patients [100]. According to the antioxidant properties of pomegranate, its peel extract was analyzed for damage related to hypertension and aging in a SHR model. Pomegranate peel extract showed a significant reduction in SBP and coronary ACE activity. The extract also reduced superoxide anion levels and vascular wall areas in the coronary SHRs treated with peel extract. Therefore, this study suggests that pomegranate peel extract may have beneficial effects on coronary heart disease [106]. Antioxidant properties related to leaf and root extracts of *Taraxacum officinale* were investigated in vitro and in vivo. For the in vivo model, experiments were performed on organ homogenate samples from L-NAME-induced Wistar rats. The leaf extract of *Taraxacum officinale* possessed significantly higher polyphenol and flavonoid, including free radical scavenging activity (EC50 0.37 compared to 1.34 mg/mL) and total antioxidant capacities (82.56% compared to 61.54% 3-ethylbenzothiazoline-6-sulfonic acid: ABTS, and 156 ± 5.28 compared to 40 ± 0.31 ferric reducing antioxidant power: FRAP). Both extracts significantly increased total antioxidant capacities (kidney and brain tissues) and reduced MDA levels (heart tissue) [115]. Taken together, there are several plants and herbs whose extracts have blood pressure-lowering properties in patients and animal models with hypertension. Therefore, these extracts could be used as a therapeutic strategy to prevent and treat hypertension-associated obesity (Table 4 and Figure 2).

## 8. Conclusions and Perspectives

Obesity and associated cardiovascular diseases have been recognized as a public health concern, mainly in countries where its prevalence is alarmingly high. According to the World Obesity Atlas 2023 estimation, 38% of the global population is currently overweight or obese. In Mexico, the projected trends in obesity prevalence (BMI ≥ 30 kg/m^2^) will be very high by 2035, 47% in adults (https://www.worldobesity.org/resources/resource-library/world-obesity-atlas-2023, accessed on 28 June 2024). Currently, various agents are used to prevent or treat obesity and associated metabolic disorders, for instance, lowering lipids (e.g., statins, inhibitors of enzyme HMG-CoA reductase) and body weight (e.g., orlistat, an inhibitor of pancreatic lipase), and common treatment strategies employed for CVDs include a combination of anticoagulant and antithrombotic therapy such as aspirin, clopidogrel (tienopyrin), apixaban, dabigatran, rivaroxban and warfarin. Unfortunately, the use of these medications causes potentially serious side effects, including nausea, vomiting, flatulence, diarrhea, insomnia, headache, hemorrhagic, and ischemic complications. For this reason, the World Health Organization (Committee, 1980) recommended the use of herbal and plant-based medicines. Compared with pharmaceutical agents, herbs and plants extract offer similar benefits against cardiometabolic risk factors associated with obesity without the side effects. Herb and plant extracts are rich sources of various nutrients and medicinal phytochemicals, including vitamins, minerals, carotenoids, fatty acids and esters, oils, polysaccharides, proteins, polyphenols, fibers, catechins, flavonoids, terpenes, and other compounds. These bioactive phytochemicals show various beneficial properties against various human diseases. For example, polyphenols have antioxidant, anti-inflammatory, antihypertensive, and atherogenic effects, and they can inhibit platelet aggregation and activation [198], thus having a potential protective role in several diseases such as obesity, T2D, and cardiovascular disease. Although most herb and plant extracts analyzed in this review were investigated in vitro and in animal models, further future research studies in clinical trials are required to confirm the beneficial properties of these herbs and plants against cardiometabolic risk factors associated with obesity. It is well known that obesity is associated with processed foods and high-calorie diets, including a sedentary lifestyle. Therefore, healthcare systems and governments in countries with a current high prevalence of obesity must encourage people to consume healthy nutrition (vegetables and fruits), physical activity, and maintain a healthy weight to avoid obesity and CVDs, which are the main cause of mortality globally. In 2021, CVDs accounted for 20.5 million deaths, of which around 80% occurred in low- and middle-income countries [199]. Therefore, overall, the consumption of herbs and plant teas should be recommended as a possible approach to reduce cardiovascular diseases associated with obesity.

## Figures and Tables

**Table 2:** Extracts from different herbs and plants targeting dyslipidemia and adipokines in obesity.

Name of Herbs and Plants and Method of Extraction	Type of Study	Doses and Duration	Outcomes and Side Effects (Humans)
*Allium sativum* (garlic), high hydrostatic pressure extract. Not available with this extract method on the market.	5-week-old male Sprague Dawley rats with HFD (45% kcal from fat)	2% (*w*/*w*) of extract for 5 weeks	Decreased in plasma TG and LDL-C levels, increased in HDL-C levels, reduced hepatic TG and TC levels, upregulated hepatic apoA-I, ABCA1, and LCAT gene expression [171]. Garlic extract table may cause breath and body odor, upset stomach or heartburn.
*Camellia sinensis*, teas (green, red, and white). Not available on the market.	3-month-old male LDLr^−/−^ mice with HFD (20% fat with 1.25% cholesterol, and 0.5% cholic acid)	25 mg/kg body weight daily for 60 days	Prevented left ventricular hypertrophy, partially prevented hyperlipidemia and insulin resistance, and reduced CRP levels [173]. Not reported side effects
*Camellia sinensis*, green tea extract (GTE). Extract on the market in different brands.	Systematic review and meta-analysis of randomized clinical trials	Varied dosages, some ≥1000 mg/day, others <1000 mg/day, and durations with subgroup analyses based on ≥12 weeks vs. <12 weeks	Significant reduced total cholesterol (TC) and LDL-C. Decreased fasting blood sugar, and HbA1c. Small increased HDL-C. Reduced diastolic blood pressure [174]. Green tea extracts can cause liver problems, and the symptoms can include yellowing of your skin or the whites of your eyes, stomach pain and nausea
Corni Fructus, extract produced by Tsumura Juntendo Inc. (Tokyo, Japan).	5-week-old male Wistar rats with a high cholesterol diet (1% cholesterol and 0.5% cholic acid)	50, 100, and 200 mg/kg/day for 10 days	Lowered blood pressure and serum cholesterol levels. Decreased atherogenic index, increased cholesterol and bile acid excretion. Reduced lipid peroxidation, up-regulated SREBP-2 and PPARα expression, and enhanced fatty acid oxidation [47]. No side effects have been reported
*Cydonia oblonga* (ethanol extract). Extract on the market in different brands.	Male Sprague Dawley rats (240 ± 20 g) induced with hyperlipidemia	Hyperlipidemia induction for 21 days and then with 40, 80, 160 mg/kg/day for 4 weeks	Reduced serum TC, TG, LDL-C, ALT, AST, increased HDL-C, reduced MDA, improved SOD and GSH-Px activity in hepatic tissues [52]. No side effects have been reported
*Ilex paraguariensis* (Yerba mate), hydroethanolic extract and n-butanolic fraction. Available on the market in different brands.	8-week-old male Wistar rats with HFD (60% kcal from fat) with cholesterol (2%) and cholic acid (0.2%)	Hyperlipidemia induction for 30 days and then with 200, 400, 800 mg/kg/day for 30 days	Reduced serum TG, cholesterol, and atherogenic index [175]. Yerba mate can cause insomnia, upset stomach, increased heart rate, and others.
*Ilex paraguariensis* (Yerba mate), aqueous extract. Available on the market in different brands.	A systematic review and meta-analysis	Various doses in included studies	No significant change in TC, LDL-C, HDL-C, and TG levels [178]. Yerba mate can cause insomnia, upset stomach, increased heart rate, and others.
*Ilex paraguariensis* (Yerba mate), aqueous extract. Available on the market in different brands.	8-week-old male Syrian golden hamsters with HFD (15% lard and 0.2% cholesterol)	Hyperlipidemia induction for 4 weeks and then with 1%, 2%, and 4% *w/v* for 4 weeks	Decreased body weight gain, lowered serum lipid levels, increased antioxidant enzyme activity, improved lipoprotein lipase (LPL) and hepatic lipase (HL) activities, and upregulated PPARα and LDL-C receptor mRNA expression. Reduced SREBP-1c and acetyl CoA carboxylase mRNA expression [176]. Yerba mate can cause insomnia, upset stomach, increased heart rate, and others.
*Ilex paraguariensis* (Yerba mate), aqueous extract. Available on the market in different brands.	Randomized clinical trial with dyslipidemic individuals	1 L/day (20 mg/mL) for 90 days	Increased serum antioxidant capacity and GSH, and decreased LDL-C [177]. Mate tea did not show adverse effects in the patients.
*Moringa oleifera* (aqueous extract).	Male Wistar rats with HFD (3% fat)	1 mg/g for 30 days	Decreased cholesterol levels in serum, liver, And kidney. Increased serum albumin [83]. *Moringa oleifera* is likely safe when the leaves, fruit, and seeds are eaten as food.
*Nigella sativa*, seed powder, seed oil, and seed (methanolic extract). Available on the market in different brands.	Systematic review of experimental and clinical studies	Variable treatment time of seed powder (100 mg–20 g daily), seed oil (20–800 mg daily), and seed extract (6, 9, 14, and 21 g/kg)	Reduced TC, LDL-C, and TG. No significant effect on HDL-C [85]. *Nigella sativa* seed can cause allergic rashes, stomach upset, vomiting, or constipation.
*Opuntia ficus-indica* (aqueous extract). Available on the mar-ket.	Triton-induced hypercholesterolemia in male Balb-c mice	500 mg/kg in a single administration for 16 h plus fasting for 8 h	Significantly decreased cholesterol levels. Inhibited pancreatic lipase with IC50 = 588.5 μg/mL [90]. *Opuntia ficus-indica* can cause nausea, bloating, mild diarrhea, increased quantity and frequency of stools, and headache.
*Platycodon* *grandiflorus* (water extract). Extract on the market in different brands.	9-week-old male C57BL/6J mice with HFD	1 g/kg/day for 8 weeks	Reduced body weight gain by 7.5%, improved plasma lipid profiles, decreased leptin, increased adiponectin, downregulated lipogenic gene expression, increased lipolysis gene expression, and inhibited adipogenic transcription factors [97]. Side effects not reported.
*Platycodon* *grandiflorus* (70% ethanol extract). Extract on the market in different brands.	5-week-old male C57BL/6J mice with HFD (40% of fat)	Dyslipidemia induction for 5 weeks and then with 25 and 75 mg/kg/day for 4 weeks	Reduced plasma and hepatic lipid levels, upregulated antioxidant proteins, inhibited oxLDL-C-induced cell death and lactate dehydrogenase release, exhibited antioxidant activity in vitro and in vivo [98]. Side effects not reported.
*Platycodon* *grandiflorus*, extract (water, 50% ethanol, and 80% ethanol). Extract on the market in different brands.	L6 muscle cells and 9-week-old male ICR mice with HFD (60% kcal from fat)	1% and 5% extract in diet for 6 weeks	Reduced food intake, body weight, epididymal fat weight, adipocyte size, and blood glucose levels. Maintained serum adiponectin, resistin, leptin, fructosamine, and triglycerides. Upregulated adiponectin mRNA, downregulated TNF-a and leptin mRNA in WAT. In L6, muscle cells increased insulin-stimulated glucose uptake [99]. Side effects not reported.

**Table 3:** Extracts from different herbs and plants targeting insulin resistance, hyperglycemia, and diabetes.

Name of Herbs and Plants and Method of Extraction	Type of Study	Doses and Duration	Outcomes and Side Effects (Humans)
*Caralluma fimbriata* (hydro-alcoholic extract). Extract on the market in different brands.	Male Wistar rats with HFD (60% of fat)	200 mg/kg/day for 90 days	Reduced hyperglycemia, hyperinsulinemia, hyperleptinemia, hypertriglyceridemia, oxidative stress, and improved insulin sensitivity [180]. *Caralluma fimbriata* can cause constipation and gas.
*Ceylon cinnamon* (hydro-alcoholic extract). Extract on the market in different brands.	Pancreatic alpha-amylase activity. 7-week-old male Wistar Han IGS rats. A randomized, placebo-controlled, cross-over clinical trial in healthy subjects.	In vitro: 0–100 µg/mL. In vivo: 6.25, 12.5, 25, 50, 100 mg/kg for 5 weeks. Humans: 1 g of extract (two 500 mg capsules), single dose post-meal.	In vitro: Inhibited pancreatic alpha-amylase (IC50 = 25 μg/mL). In vivo: Reduced glycemic response to starch. Human: Lowered postprandial glycemia by 14.8% (0–120 min) and 21.2% (0–60 min) without increasing insulin secretion [30]. No side effects were reported during the study.
*Citrullus colocynthis* (petroleum ether, water or 80% methanol, ethyl acetate, and n-butanol, crude aqueous extracts). Extract on the market in different brands.	3T3-L1 adipocytes	4, 20, or 100 μg/mL for 24, 48, and 96 h	Enhanced insulin-induced GLUT4 translocation and glucose uptake, and increased insulin-induced PKB phosphorylation [181]. Colocynth can cause severe irritation of the stomach and intestine lining, bloody diarrhea, bloody urine, kidney damage, and inability to urinate. Also, can cause convulsions, paralysis, and death.
*Citrullus colocynthis * (tablets, capsules, or oral drops). Not available on the market.	Randomized Controlled Clinical Trials	Different doses for 30 to 60 days	No significant effect on FBS, HbA1c, LDL-C, TC, and TG. Increased HDL-C levels [33]. No serious side effects of this plant were reported.
Corni Fructus (water extract). Not available on the market as extract.	7-week-old male C57BUKsJ-db/db mice and C57BL/6 mice	500 mg/kg/day for 8 weeks	Reduced blood glucose levels, improved insulin resistance, and increased glucose utilization [48]. No side effects have been reported.
Corni Fructus (aqueous extract). Extract on the market in different brands.	7-week-old male C57BL/KsJ-db/db mice	500 mg/kg/day for 8 weeks	Reduced oxidative stress, increased SOD activity, decreased XO, CAT, and GST activities. Lower mRNA expression of eNOS in kidneys [182]. No side effects have been reported.
*Ginkgo biloba * (aqueous and 12% ethanol extracts). Extract on the market in different brands.	α-amylase and α-glucosidase activities	10, 25 and 50 mg/mL of Ginkgo leaf extract	Aqueous extracts had higher total phenolic content but only ethanolic extracts inhibited ACE, a strong correlation between total phenolics and α-glucosidase inhibitory activity, and to a lesser degree positive correlation between total phenolics and α-amylase inhibitory activity [57]. *Ginkgo biloba* can cause stomach upset, headache, dizziness, and allergic skin reactions. *Ginkgo* leaf extract might increase the risk of bruising and bleeding or cause arrhythmia.
*Ginkgo biloba* (Egb761). Available on the market.	RAoSMCs and HUVECs. Five-week-old male Otsuka Long-Evans Tokushima Fatty rats and five-week-old male ApoE^−/−^ mice	Obesity and insulin resistance induction for 24 weeks (rats). Two months in all mice with HFD (42% fat, 1.25% cholesterol). All animals with 100 mg/kg and 200 mg/kg for 6 weeks (rats), and 2 months (mice).	Reduced intima–media ratio. Induced greater apoptosis in rats, improved glucose homeostasis and increased circulating adiponectin levels, decreased plasma hs-CRP concentrations. In vitro: Decreased VSMC proliferation and migration, Increased caspase-3 activity and DNA fragmentation, decreased monocyte adhesion and ICAM-1/VCAM-1 levels. Kaempferol and quercetin: Reduced VSMC migration and increased caspase activity and protect against atherosclerosis [58]. Ginkgo biloba can cause stomach upset, headache, dizziness, and allergic skin reactions. Ginkgo leaf extract might increase the risk of bruising and bleeding or cause arrhythmia.
Green coffee (*Coffea*), aqueous extract). Extract on the market in different brands.	A randomized, double-blind, placebo-controlled trial	400 mg (capsules) twice per day for 10 weeks	Decreased SBP, TG, hs-CRP, increased HDL-C, and marginally significant reduction in FBG. No significant changes in DBP, LDL-C, TC, insulin levels, HOMA-IR, and MDA [183]. Consuming large amounts of green coffee might cause headache, anxiety, agitation, and irregular heartbeat.
*Hibiscus sabdariffa*, (polyphenolic extract by methanol). Extract on the market in different brands.	8-week-old male Sprague Dawley rats with HFD and STZ	Type 2 diabetes induction: HFD for 7 weeks and then HFD and STZ for 2 weeks. Doses 100 mg/kg and 200 mg/kg for 7 weeks.	Reduced hyperglycemia, hyperinsulinemia, serum TG, cholesterol, and LDL-C/HDL-C ratio. Decreased plasma AGE formation and lipid peroxidation. Inhibited CTGF and RAGE expression in aortic regions. Improved weight loss in diabetic rats [184]. *Hibiscus sabdariffa* can cause stomach upset, gas, and constipation.
*Hibiscus sabdariffa* (aqueous extract). Extract on the market in different brands.	α-amylase and α-glucosidase activities	Red and white varieties; IC50 values: 25.2 µg/mL (red) and 47.4 µg/mL (white) for α-glucosidase inhibition; 90.5 µg/mL (white) and 187.9 µg/mL (red) for α-amylase inhibition	Both varieties inhibited α-amylase and α-glucosidase activities, red variety exhibited higher α-glucosidase inhibitory activity, while the white variety showed higher α-amylase inhibitory activity, and strong antioxidant properties, particularly in the red variety [70]. *Hibiscus sabdariffa* can cause stomach upset, gas, and constipation.
*Hibiscus sabdariffa* (aqueous extract). Extract on the market in different brands.	3T3-L1 cells and male Sprague Dawley rats (100–120 g) with HFD	In vitro: 0.1, 0.5, 1 mg/mL. In vivo: 250 and 500 mg/kg/day for 8 weeks	Reduced body weight, food intake, lipid profiles, inflammatory cytokines, lipid peroxidation, serum leptin, insulin, and duodenal glucose absorption. Increased glucose uptake in adipose tissue and muscle, downregulated adipogenic Gene expression [69]. *Hibiscus sabdariffa* can cause stomach upset, gas, and constipation.
*Ilex paraguariensis* (Yerba mate), aqueous extract. Extract on the market in different brands.	T2DM and pre-diabetes subjects	330 mL of roasted mate tea 3 times a day for 60 days	T2DM: Significant decrease in fasting glucose, HbA1c, and LDL-C. Pre-diabetes: Significant decrease in LDL-C, non-HDL-C, and TG. Improved glycemic control and lipid profile, reduced risk of coronary disease [77]. Mate tea did not show adverse effects in the patients.
*Moringa oleifera* (dry leaf powder). Available on the market in different brands.	A double-blind, randomized, placebo-controlled, parallel-group clinical trial	2400 mg/day (6 capsules/day) for 12 weeks	Significant decrease in FBG and HbA1c. No significant changes in microbiota, hepatic and renal function markers, or appetite-controlling hormones [84]. *Moringa oleifera* has no side effects with supplementation [84].
*Opuntia ficus-indica* var. *saboten* (hot-water extract). Extract on the market in different brands.	α-Glucosidase activity. L6 muscle cells. 5-week-old male C57BL/6J db/db mice and their non-diabetic heterozygous littermates (db/-), and 9-week-old male ICR mice	α-Glucosidase activity (1, 5, 10 mg/mL). L6 muscle cells (1–200 µg/mL). db/db mice (1 and 2 g/kg BW) and db/- mice (1 g/kg BW) for 4 weeks.	Inhibited α-glucosidase activity and intestinal glucose absorption. In L6 muscle cells, increased glucose uptake, stimulated AMPK and p38 MAPK phosphorylation, and increased GLUT4. In db/db mice, improved hyperglycemia, hyperinsulinemia, glucose tolerance, and regenerated β-cells [185]. *Opuntia ficus-indica* var. *saboten* had no adverse side effects on normal mice [185].
*Opuntia ficus-indica* (cladodes and stem/fruit skin-blend ratio 75/25) hot-water extract. Not available combined on the market.	Wistar rats either sex weighing 250–350 g	0.176–176 mg/kg for 180 min and glucose (i.p., 2 g/kg in 5 mL) 30 min after extracts administration	Both extracts lowered blood glucose levels (in doses as low as 6 mg/kg). The blend increased basal plasma insulin levels [92]. No side effects have been reported for *Opuntia ficus-indica* (cladodes and stem/fruit skin).
*Punica granatum* (methanolic extract). Extract on the market in different brands.	α-glucosidase activity assay. Zucker diabetic fatty (ZDF) rats and Zucker lean (ZL) rats (14–15 weeks old).	α-glucosidase activity (200 µL of extract for 5 min). 500 mg/kg body weight, oral in 5% acacia once daily for 2 weeks.	Lowered plasma glucose levels in non-fasted ZDF rats, inhibited postprandial hyperglycemia, potent inhibitory effect on α-glucosidase activity (IC50: 1.8 µg/mL) [105]. Some people have experienced sensitivity to pomegranate extract such as itching, swelling, runny nose, and difficulty breathing.
*Salvia miltiorrhiza* (water extract). Extract on the market in different brands.	HMEC-1 cells	10 µg/mL of extract in 30 mM glucose condition for 48 h	Decreased VEGF mRNA and ROS formation induced by high glucose, and UCP-2 siRNA abolished these effects [109]. *Salvia miltiorrhiza* can cause upset stomach, itching, and reduced appetite.
*Salvia miltiorrhiza* (different extracts). Extract on the market in different brands.	Review of preclinical and clinical studies on diabetes and complication	Not applicable	SM exhibits antidiabetic activities, including anti-inflammation, antioxidation, antifibrosis, and antiapoptosis. Key pathways involved are Wnt/β-catenin, TSP-1/TGF-β1/STAT3, JNK/PI3K/AKT, and others. The main compounds include salvianolic acids and diterpenoid tanshinones [107]. *Salvia miltiorrhiza* can cause upset stomach, itching, and reduced appetite.
*Taraxacum officinale* (aqueous extract). Extract on the market in different brands.	α-amylase and α-glucosidase activities	1, 10, 20, 30 mg/mL	Shade-dried leaves demonstrated potent antidiabetic activity via inhibiting α-amylase and α-glucosidase in a dose-dependent manner [116]. *Taraxacum officinale* can cause allergic reactions, stomach discomfort, diarrhea, or heartburn in some people.

**Table 4:** Extracts from different herbs and plants with antihypertensive effects.

Name of Herbs and Plants and Method of Extraction	Type of Study	Doses and Duration	Outcomes and Side Effects (Humans)
*Allium sativum * (aged black garlic extract). Available on the market in different brands	Randomized, crossover, double-blind, sustained, and controlled study; individuals with moderate hypercholesterolemia	250 mg (1.25 mg SAC)/tablet/day ABG for 6 weeks, with 3 weeks of washout	Significantly decreased DBP, particularly in men with a baseline DBP higher than 75 mm Hg and improved cardiovascular risk factors [188]. Garlic extract table may cause breath and body odor, upset stomach or heartburn.
*Andrographis* *paniculata * (aqueous extract). Extract on the market in different brands	Male SHR and WKY rats, aged 14–15 weeks	2.8, 1.4, 0.7 g/kg for 13 days	Lowered SBP in SHR and WKY rats, reduced plasma ACE activity and kidney TBA level in SHR. No significant effect on lung ACE activity [189]. *Andrographis* can cause side effects diarrhea, vomiting, rash, headache, runny nose, and fatigue
*Aronia melanocarpa* (chokeberry), berry extracts. Available on the market in different brands	Meta-analysis of controlled clinical trials, including randomized, placebo-controlled trials	Daily supplementation for an average of 6–8 weeks	Significantly reduces systolic blood pressure and TC, with stronger effects in adults over the age of 50 years [190]. Adverse effects were not found. Taking chokeberry together with drugs that slow blood clotting might increase the risk of bruising and bleeding
*Camellia sinensis* (Green tea), green tea extract (GTE). Extract on the market in different brands	Crossover, randomized, double-blind, placebo-controlled clinical trial	Three capsules daily, each containing 500 mg of GTE (260 mg polyphenols per capsule), for 4 weeks with a 2-week washout period between treatments	Significant decrease in SBP at 24 h, daytime, and nighttime in obese prehypertensive women. No significant changes in DBP or other metabolic parameters [193]. Green tea extracts can cause liver problems, and the symptoms can include yellowing of the skin or the whites of the eyes, stomach pain and nausea
*Camellia sinensis* (Green Tea), green tea extract (GTE). Extract on the market in different brands	13-week-old male Sprague Dawley rats	High dose (700 g/kg/day) or low dose (350 g/kg/day) Ang II dose for 13 days, 6 mg/mL GTE in drinking water	GTE prevented hypertension, left-ventricular hypertrophy, vascular remodeling, and endothelial dysfunction induced by high Ang II dose. It blunted increases in oxidative stress markers [191]. Green tea extracts can cause liver problems, and the symptoms can include yellowing of the skin or the whites of the eyes, stomach pain and nausea
*Camellia sinensis* (Green tea), green tea extract (GTE). Extract on the market in different brands	A systematic review and meta-analysis of randomized clinical trials	Various doses and durations across multiple studies	Green tea epigallocatechins have ACE inhibitor properties. Green tea lowers blood pressure by suppressing NADPH oxidase activity and reducing reactive oxygen species. Some meta-analyses reported beneficial effects on blood vessel dilation and lipid profile [192]. Green tea extracts can cause constipation, abdominal discomfort, hypoglycemia, elevated BP, dyspepsia, and mild skin rash
Cocoa (flavanols-rich cocoa extract). Supplied by Nutrafur S.A. (Murcia, Spain)	Clinical trial, crossover, randomized, double-blind	1.4 g of cocoa extract (415 mg flavanols) daily for 4 weeks	Reduced postprandial SBP after daily cocoa extract intake within an energy-restricted diet [41]. Cocoa can cause allergic skin reactions, migraine headaches, nausea, stomach discomfort, constipation, and gas. Eating large amounts can cause caffeine-related side effects such as nervousness, increased urination, sleeplessness, and a fast heartbeat
*Ginkgo biloba * (Standardized leaf extract, Egb761). Available on the mar-ket	Male adult Wistar rats (120–160 g), hypertension induced by L-NAME and hypercholesterolemia induced by 1% cholesterol diet	100 mg/kg/day orally for 12 weeks	Reduced systolic, diastolic, and mean arterial BP. Improved serum lipid profile, protected against renal injury, reduced renal oxidative stress, nitrosative stress, and inflammation. Decreased renal TNF-α, IL-6, IL-1β, and iNOS protein expressions, and increased eNOS protein expression [59]. *Ginkgo biloba* can cause stomach upset, headache, dizziness, and allergic skin reactions. *Ginkgo* leaf extract might increase the risk of bruising and bleeding or cause arrhythmia
*Ginkgo biloba* (new component group of *Ginkgo biloba* leaves, GBLCG), 50% ethanol extract. Available as leaves extract in the market	Male Wistar rats and spontaneously hypertensive rats (SHRs), 200 ± 20 g	4.4, 2.2, and 1.1 mg/kg for 120 days	Reduced blood pressure and improved myocardial hypertrophy by promoting NO synthesis and release in endothelial cells, reducing oxidative stress, inhibiting platelet aggregation, and promoting lesion circulation. The hypotensive activity of GBLCG (4.4 mg/kg) was better than *Ginkgo biloba* extract [194]. *Ginkgo biloba* can cause stomach upset, headache, dizziness, and allergic skin reactions. *Ginkgo* leaf extract might increase the risk of bruising and bleeding or cause arrhythmia
*Coffea* (green coffee bean extract, GCE) hot-water extract. Extract on the market in different brands	Healthy male volunteers (aged 30 to 50 years), with mild hypertension	46 mg, 93 mg, or 185 mg of GCE daily for 28 days	Dose-dependent reduction in SBP. Reduction in DBP was also observed [64]. Consuming large amounts of green coffee might cause headache, anxiety, agitation, and irregular heartbeat
*Hibiscus sabdariffa* (dried calyx and hibiscus anthocyanins), water extract. Available as dried calyces in the market	12-week-old male SHR	10%, 15%, and 20% *Hibiscus sabdariffa* for 10 weeks. 50, 100, and 200 mg/kg red anthocyanin by oral gavage for 5 days	*Hibiscus sabdariffa* reduced SBP, DBP, and LV mass; increased myocardial capillary surface area and length density. Red anthocyanin did not significantly reduce the SBP and DBP [195]. *Hibiscus sabdariffa* can cause stomach upset, gas, and constipation
*Hibiscus sabdariffa* (aqueous extract). Extract on the market in different brands	Wistar, Wistar-Kyoto (WKY), and SHR of about 16 weeks old	SHR (EC50 = 0.83 ± 0.08 mg/mL), WKY (EC50 = 0.46 ± 0.04 mg/mL), and Wistar rats (EC50 = 0.44 ± 0.08 mg/mL)	Concentration-dependent relaxant effect on mesenteric arteries and reduced L-type calcium current [71]. *Hibiscus sabdariffa* can cause stomach upset, gas, and constipation
*Hibiscus sabdariffa* calyces (HSC), aqueous extract of calyces. Available as dried calyces in the market	A randomized, controlled, single-blinded, acute, cross-over trial	7.5 g HSC in 250 mL Buxton water, at time 0 min followed by a medium fat lunch at 120 min in a random order separated by a two-week washout period	Significant increase in % flow-mediated dilatation, non-significant decrease in SBP and DBP, non-significant increase in urinary and plasma NOx, the reduced response of serum glucose, plasma insulin, serum TAG, and CRP levels. Significant improvement in systemic antioxidant response. No significant changes in arterial stiffness [197]. *Hibiscus sabdariffa* can cause stomach upset, gas, and constipation
*Hibiscus sabdariffa* (aqueous extract). Extract on the market in different brands	Double-blind randomized controlled trial	150 mg/kg daily for 4 weeks	Reduced plasma aldosterone, serum ACE, and increased plasma renin activity [196]. *Hibiscus sabdariffa* can cause stomach upset, gas, and constipation
*Nigella sativa * (seed), boiled water extract. Available on the market	A randomized, double-blind, placebo-controlled trial. Healthy male volunteers with mild hypertension	100 mg and 200 mg twice a day for 8 weeks	Significant reduction in SBP and DBP in a dose-dependent manner. Reduced TC and LDL-C levels [87]. *Nigella sativa* seed can cause allergic rashes, stomach upset, vomiting, or constipation
*Platycodon grandiflorus* (roots) (aqueous extract). Available on the market	H9c2 myoblasts. SHRs and WKYs rats (about 300 g)	1.25, 2.5, 5 µg/µL for in vitro. 100 and 200 mg/kg/day for 50 days for in vivo	Suppressed Ang II-induced IGF-IIR signaling, reduced cardiomyocyte apoptosis, decreased SBP and DBP in SHRs [100]. Side effects not reported
*Punica granatum* (pomegranate peel), ethanol (95° GL) Extract. Available in the market as pomegranate peel powder	Female SHRs (4 and 28 weeks old)	25 mg/100 g rat for 30 days	Reduced SBP, coronary ACE activity, oxidative stress, and vascular remodeling in hypertensive female rats [106]. Some people have experienced sensitivity to pomegranate extract such as itching, swelling, runny nose, and difficulty breathing
*Taraxacum officinale* (leaves and roots), 70% ethanol extract. Available on the market	ABTS and FRAP. L-NAME-induced hypertensive Wistar rats (150 g to 200 g), both sexes	500 mg/kg/day for 21 days	Leaves possessed higher polyphenol and flavonoid, free radical scavenging activity, and total antioxidant capacities. Leaves and roots extract significantly increased total antioxidant capacities (kidney and brain tissues) and reduced MDA levels (heart tissue) [115]. *Taraxacum officinale* can cause allergic reactions, stomach discomfort, diarrhea, or heartburn in some people

## Data Availability

Data sharing is not applicable.
